# Preparation and Characterization of Calcium Cross-Linked Starch Monolithic Cryogels and Their Application as Cost-Effective Green Filters

**DOI:** 10.3390/polym13223975

**Published:** 2021-11-17

**Authors:** Chanita Boonkanon, Kharittha Phatthanawiwat, Laemthong Chuenchom, Nareumon Lamthornkit, Tarawee Taweekarn, Worawit Wongniramaikul, Aree Choodum

**Affiliations:** 1 Integrated Science and Technology Research Center, Faculty of Technology and Environment, Prince of Songkla University, Phuket Campus, Kathu, Phuket 83120, Thailand; chanitanbkn@gmail.com (C.B.); kharittha.p@gmail.com (K.P.); jar.nareumon@gmail.com (N.L.); T.tarawee@hotmail.com (T.T.); worawit.won@phuket.psu.ac.th (W.W.); 2 Division of Physical Science and Center of Excellence for Innovation in Chemistry, Faculty of Science, Prince of Songkla University, Hat Yai Campus, Hat Yai, Songkhla 90110, Thailand; laemthong.c@psu.ac.th

**Keywords:** starch, limewater, biodegradable cryogel, biodegradable filter, macropore

## Abstract

Monolithic cryogels from starch were successfully synthesized and applied as alternative biodegradable filters for the first time. Rice flour was cross-linked with Ca^2+^ from limewater during gelatinization before being frozen and then thawed for three cycles. The resultant material was then soaked in ethanol for 3 h before incubation at 80 °C for 1 h, yielding monolithic material with interconnected pores in sizes of 51 ± 18 to 52 ± 15 µm without any need of freeze-drying. The cryogels possessed macroporous structure with specific surface areas from 1.1 to 4.3 m^2^g^−1^, they could adsorb water from 599 ± 27 to 635 ± 59% of their dry weight with low swelling ratios of 6.0 ± 0.3 to 6.4 ± 0.6 g_water_/g_cryogel_, and could be applied as biofilters to remove suspended particles and reduce the light absorption of water sample from 25 ± 3 to 96 ± 5%. The prepared biofilters can be re-used up to three times, although they cost only USD 0.0004/piece. Complete weight loss resulted from burial in soil for 30 days, indicating environmentally friendly biodegradation and potential for environmental applications.

## 1. Introduction

Cryogels are cryogenically-structured polymeric materials, and have been used in biomedical [1,2], cell and tissue engineering [3], and environmental [4] applications; they are macroporous materials with a three-dimensional flexible structure, allowing for the effective mass transport of macromolecular solutes or migration of cells with high biocompatibility [5,6]. High water retention capacity can also be achieved, which is beneficial for aqueous phase applications. This type of material also has open-ended pores interconnected to form flow channels that facilitate fast diffusion, resulting in a fast response without any limitations or backpressure even in a high-flow-rate application [4]. Cryogels are also highly elastic and strong even under large deformations. They can be repeatedly compressed by at least 50% from their initial volume, without serious damage to the porous structure, because of their good mechanical stability [6,7,8].

Cryogels are synthesized from appropriate monomers or polymeric precursors via a freeze thawing technique (known as cryogelation, cryotropic gelation, or cryostructuration [5,6,9,10]). The gel precursors (monomers, cross-linkers, and initiators) induce either physical or chemical crosslinking in semi-frozen liquid medium at temperatures between −5 °C and −20 °C, wherein the ice crystals act as porogens and thus as templates for the shape and size of the interconnected pores seen after thawing [6], i.e., the mixture consists of two main parts during freezing: the ice crystals, and a liquid microphase between the ice crystals. The chemical reaction (cross-linking) takes place in the non-frozen liquid microphase [11], resulting in the formation of relatively thin nano/microporous walls [9,12,13], while the ice crystals continue to grow and merge with other crystals until a complete frozen frame of interconnected ice crystals is formed. The growth of the ice crystals can cause significant pressure on the formed pore walls typically making the walls relatively thin with low nano/microporosity [5]. Thus, the main stage of cryogel production is in the frozen state with gelation (cross-linking) at temperatures between −5 and −20 °C. The gelation temperature affects the pore size distribution of the obtained cryogel, while the type and composition of the cross-linker affect the swelling degree of the material [6]. The freezing rate can also affect the final properties, i.e., a slower rate tends to give larger pores with increased interconnectivity, while a faster freezing rate produces mechanically weaker cryogel with a low level of interconnectivity due to supercooling of the solvent before ice crystals begin to form, yielding small, irregular pores [14]. Changes in the amount of water and organic co-solvent, as well as ionic strength, pH, freeze thaw cycle, other precursor components, and polymerization conditions, also cause significant variations in the structural characteristics of the obtained cryogel [12,13]. However, the pore size has been reported to be the property most affected by these changes [10]. After having been frozen for an appropriate time, the material is allowed to thaw at room temperature, making the ice crystals melt and leaving an interconnected macroporous structure that is sponge-like and mechanically stable. The total pore volume of the native cryogels can be huge, up to 20–40 cm^3^/g [9,12,13], while the macropore walls (typically 1–10 µm, rarely 15–50 µm in thickness) can include both nanopores (d_nano_ < 0.1 µm) and micropores (d_micro_ > 0.1 µm) [5].

Various polymeric precursors have been reported for cryogel preparation [3,5,6]. Both natural and synthetic polymers can be used to produce cryogels with different morphologies and porosities [3,5,6]. Synthetic polyvinyl alcohol (PVA) is the first and the most popular polymer that has been used for cryogel preparation, both alone and in combination with other polymers [5,15,16]. This is because its structure containing (-CH_2_-C(OH)H-)_n_ chains, allows for various degrees of cross-linking with the participation of the active COH groups, along with various cross-linkers and co-polymers. Other synthetic polymers have also been reported, such as poly(vinyl pyrrolidone) (PVP) [15], poly(4-vinyl pyridine) [17], and Jeffamine diamino hexane [18]. Natural polymers are sometimes a good choice as they tend to have low toxicity and good biocompatibility. Proteins, e.g., collagen and gelatin, are popular precursors for the preparation of cryogels because of their great biocompatibility and significant functionalization. Spongy collagen and gelatin cryogels can be prepared via cross-linking with dialdehyde starch [19,20,21,22,23]. Biocompatible polysaccharides and related compounds, e.g., chitosan or hyaluronic acid, have also been reported [24,25,26]. They have numerous hydroxyls and other functionalities with O and N atoms similar to PVA, but with more complex structure of cyclic monomers. Agarose-alginate cryogels have also been synthesized in different shapes such as monoliths, sheets, discs, and beads with mechanical strength similar to soft natural tissues [27]. Physically and chemically cross-linked cellulose cryogels have also been reported using epichlorohydrin as a cross-linker [28]. PVA has also been combined with microcrystalline cellulose [29]. Starch is one of the most promising precursors for cryogel preparation because of its low cost, abundant supply, good processability, biodegradability, and ease of chemical modifications [30,31]. It is commonly used together with synthetic polymers, e.g., N,N-dimethylaminoethyl methacrylate [30], glycidyl methacrylate [32] and polyacrylamide [31,33] due to a lack of functional groups for cross-linking [32]. Recently, cryogels from waxy starch have been prepared without any additional synthetic polymers, however freeze drying at −82 °C [34] or at −150 °C [35] was required.

In this work, an alternative procedure for the greener preparation of macroporous monolithic (single-rod) cryogels using starch is reported. The procedure to prepare the Thai dessert “Lod-chong” was modified to prepare a novel single-rod cryogel from rice flour without any additional synthetic polymers. The gel precursor was prepared via gelatinization of rice flour in the presence of calcium ions from alkaline limewater (calcium hydroxide solution commonly used as a functional ingredient to give cohesiveness and firmness to traditional desserts [36]) that act as cross-linkers. The gelatinized starch was then subjected to a conventional freeze thaw technique (at −18 °C) instead of freeze-drying at −82 °C or at −150 °C as reported previously [34,35] to produce the cryogel. The morphology, porosity, water uptake capability, water retention, and biodegradability of the prepared cryogels were tested. The novel cryogels prepared via the alternative procedure reported in this work for the first time were then applied as filters for water samples with satisfactory preliminary results.

## 2. Materials and Methods

### 2.1. Materials

Rice flour (Erawan Brand, Nakhon Pathom, Thailand) was locally purchased from a supermarket; its amylose content was analyzed using a spectrophotometer, with reference to Thai agriculture standard TAS 4000-2003 (in-house method TE-PH-021, based on Quality and Testing of Thai Horm Mali Rice, 2004, Department of Agriculture, Bangkok, Thailand), and 23.3% amylose was found. Red lime (RL) and white lime (WL) (no brand, food grade) were also bought from a supermarket in Phuket, Thailand. It is worth noting that WL is calcium hydroxide, which is commonly prepared by adding water to CaO, while RL is the mixture of WL with curcumin. Calcium hydroxide was supplied by ITW Reagents (Darmstadt, Germany). Ethanol (95%), hydrochloric acid, and sodium hydroxide were purchased from Merck (Darmstadt, Germany). Ultrapure water was obtained using a water purification system (Merck, Darmstadt, Germany).

### 2.2. Preparation of Cryogels

Calcium hydroxide solutions were prepared both in the form of limewater, which is commonly used in the preparation of Lod-chong noodles, and standard calcium hydroxide solution. Various compositions of calcium hydroxide solution were prepared by dissolving 0.08, 0.16, 0.24, and 0.32%*w/v* of red lime (RL), white lime (WL), and calcium hydroxide standard (Ca(OH)_2_) in ultrapure water as discussed in the Supplementary Materials (Figures S1–S4). The concentrations of Ca^2+^ in the solution is reported in Table 1.

Rice flour (4, 8, 12, and 16 g) was dispersed in 60 mL of prepared limewater (clear solutions from RL and WL) as well as prepared Ca(OH)_2_ solution and heated (gradually increasing from 70 to 90 °C for 30 min) until the starch was completely gelatinized and became clear. The gelatinized starch was cooled to room temperature under continued stirring for 5 min. The homogeneous mixture was filled into 3 mL plastic syringes that were placed in a freezer at −18 °C for 24 h. The resultant monolith was then thawed at room temperature, and the freeze thaw cycles were appropriately repeated (for 1–7 cycles). The obtained single-rod cryogel was then removed from the container and cut into smaller pieces (~1 cm in length); they were soaked in 95% ethanol for an appropriate time (1, 2, 3, 6, 12, 18, and 24 h) before incubation in an oven at an appropriate temperature (60, 80, and 100 °C) and for various times (20, 40, 60, and 90 min). All dried cryogels were stored in zip-lock plastic bags and placed in a desiccator until further use.

### 2.3. Characterization of Cryogels

The morphology of the cryogels was investigated using field emission scanning electron microscopy (FE-SEM; FEI, Brno, Czech Republic). The samples were sputter coated with a thin film of gold under vacuum before imaging. The functional groups of cryogels, red lime paste, and white lime paste were analyzed with Fourier transform infrared spectroscopy (FT-IR; Bruker, Berlin, Germany) using KBr pellet and ATR technique at 4000–600 cm^−1^. X-ray diffraction (XRD) patterns were analyzed with X-ray diffractometer (Empyrean, Panalytical, Almelo, Netherlands) under monochromatic Cu Kα radiation. The surface area and porosity were determined from nitrogen adsorption/desorption isotherms using a high throughput surface area and porosity analyzer (ASAP2460, Micromeritics, GA, USA) at 77 K. The prepared cryogels were degassed at 105 °C for 30 min to remove physically absorbed gases from the sample surface before analysis. The specific surface area (S_BET_) was estimated using the Brunauer–Emmet–Teller (BET) method, while the pore volume was obtained from determination using P/P_0_ of 1. The average pore diameter was calculated using the Barrett-Joyner-Halenda (BJH) method.

### 2.4. Swelling Ratio, Water Uptake Capacity, Water Retention, and Porosity of Cryogels

The swelling ratio and water retention of cryogels were investigated as described in prior reports [37,38]. The swelling ratio was calculated by weighing the dried and wet cryogels. Three completely dried cryogels of similar size (0.8 mm × 1 cm length) and weight (~0.1 g) were equilibrated in 30 mL of ultrapure water at ambient temperature. The water-absorbed cryogels were weighed after removing the surface excess water with filter paper at certain time intervals up to equilibrium (1 to 60 min). The swelling ratio (S_g_/_g_) was calculated using Equation (1) [37,38], where W*_t_* is the weight of swollen cryogel at the time of observation and W*_0_* is the weight of dried cryogel. The swelling behavior of the cryogel was analyzed at pH values ranging from 2 to 13, and at various temperatures (20, 30, 40, 50, and 60 °C).

(1) $$\mathrm{Swelling}\;\mathrm{ratio}\;(S_{g/g})=\frac{\left(W_{t}-W_{0}\right)}{W_{0}}$$

The water uptake capacity (%) was calculated using Equation (2) [37,38] where W*_e_* is the weight of swollen cryogel at equilibrium. The 24-h water uptake capacity was also determined by immersing the cryogel in distilled water at room temperature for 24 h. The average weights of three cryogels before (W_0_) and after testing (W*_24-h_*) were investigated, and the percentage of water uptake capacity (Absorbency) was calculated using Equation (3) [33]:

(2) $$\mathrm{Water}\;\mathrm{uptake}\;\mathrm{capacity}\;(\%)=100\times \frac{\left(W_{t}-W_{0}\right)}{\left(W_{e}-W_{0}\right)}$$

(3) $$\mathrm{Absorbency}\;(\%)=100\times \frac{\left(W_{24-h}-W_{0}\right)}{W_{0}}$$

The equilibrated cryogels were then put in Petri dishes at room temperature and re-weighed at specific times. The weights of the cryogels (W*_T_*) were recorded during the course of deswelling until they had reached steady levels. The water retention (%) can be calculated using Equation (4) [37]:

(4) $$\mathrm{Water}\;\mathrm{retention}\;(\%)=100\times \frac{\left(W_{T}-W_{0}\right)}{\left(W_{e}-W_{0}\right)}$$

The porosity of the cryogels (%) was also determined by squeezing the swollen gels, using Equation (5) [6]. The weight of swollen gel (W*_e_*) was compared to the weight after squeezing (W*_q_*).

(5) $$\mathrm{Porosity}\;(\%)=100\times \frac{\left(We-Wq\right)}{We}$$

### 2.5. Application of Cryogels as Biofilters

The 0.32% RL and 0.16% Ca(OH)_2_ cryogels were preliminarily tested as biofilters for four surface water samples randomly collected from Phuket, Thailand and one soil extract available in the laboratory. The ultrapure water (4 mL) was first loaded with gravity flow through the biofilters before loading a 4 mL water sample. The light absorption spectra of each sample were scanned (200–800 nm) before and after filtration for comparison. The filters were then cleaned by flushing in the reverse flow direction, before being washed with ultrapure water (10 mL) and then re-used to test their reusability.

## 3. Results and Discussion

### 3.1. Preparation of Cryogels

Monolithic cryogels based on native starch were synthesized by modifying the procedure for the preparation of Lod-chong as shown in the Supplementary Materials (Figure S5). When calcium hydroxide solutions (pH ~12) were mixed with starch and gelatinized, the pH decreased in all cases of calcium hydroxide solutions (pH ~11; Supplementary Materials, Figure S3) due to the binding between Ca^2+^ or Ca(OH)^+^ and flour [39]. After stopping the heating but still mixing with the starch, the pH of the mixture reached its maximum at concentrations higher than those for the solutions. Apparently, some hydroxyl groups of starch were oxidized at high pH making sites for interactions with the Ca^2+^ or Ca(OH)^+^ from dissociated Ca(OH)_2_, thereby shifting the equilibrium and allowing for greater solubilization of lime [39,40]. Thus, the results indicate that limewater acts as a cross-linker for ionic cross-linking of starch in a gelatinized state and this tightens the starch chains [41].

**Figure 1:**
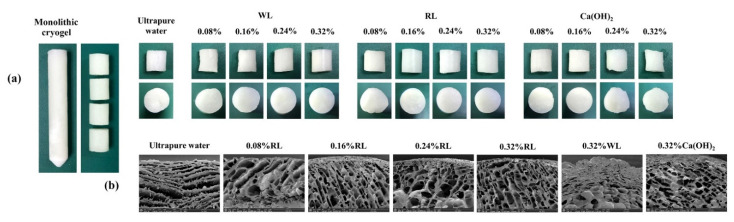
(**a**) The cryogels obtained with various amounts of red lime (RL), white lime (WL) and calcium hydroxide standard solution (Ca(OH)_2_) prepared using the limewater on the day of its preparation with one freeze–thaw-cycle, and (**b**) their FE-SEM images.

The obtained single-rod macroporous material (6 cm in length) can be cut to the desired length for further application (Figure 1a). Although the cryogel from ultrapure water was similar in appearance to all types of calcium hydroxide solution, it was damaged after applying compression matching previous reports stating that better elasticity and plasticity have been found for the starch gel with Ca^2+^ due to the effects of Ca^2+^ on the physicochemical properties of starch [42]. Without Ca^2+^ ions in acidic condition (pH 5.4 to 5.5), no ionic cross-linking occurred resulting in a weaker gel; thus, the ice crystals could easily push aside the pore walls to connect with nearby crystals, thus leaving larger pores oriented in the direction of the ice growth after cryogenic treatment [43], as shown in Figure 1b.

**Figure 2:**
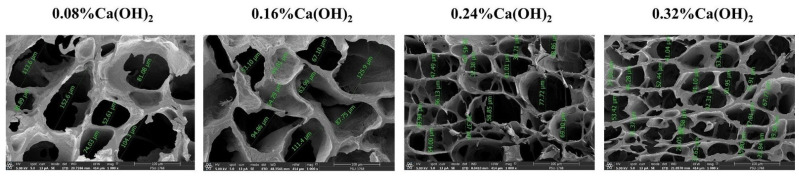
FE-SEM images of the cryogels prepared using various amounts of Ca(OH)_2_ in limewater prepared at least three days earlier and with three freeze thaw cycles.

The cryogel prepared with 0.08% RL was damaged after compression, while preparation with a larger amount produced more resilient samples. Increased porosity and swelling ratio were obtained when increasing %RL from 0.16% to 0.32%, as shown in Table 1. For limewater from WL, the cryogels synthesized with 0.08–0.24% WL were easy to damage by compression, and only the 0.32% WL case recovered its shape and had similar porosity and swelling ratio to 0.32% RL although the concentration of calcium ions in 0.32% WL was lower than in 0.32% RL (Table 1). The results indicate that not only Ca^2+^ ions play a role in cross-linking. In the case of cryogels prepared using standard calcium hydroxide solution, increasing the amount of Ca(OH)_2_ from 0.08% to 0.32% led to smaller pore size in the cryogels (Figure 2) due to the higher amount of Ca^2+^ providing more ionic cross-linking, producing a stronger network that could tolerate the pressure from ice crystals during freezing. However, the porosity of the 0.24% and 0.32% Ca(OH)_2_ cases was below that of those prepared with a lower amount (0.08% or 0.16%), and the same was true of the swelling ratios (Table 1). The cryogels from 0.08% and 0.16% Ca(OH)_2_ could also recover their shape better, indicating higher elasticity than in the 0.24% and 0.32% cases; this may be due to a greater degree of cross-linking at higher concentration of calcium hydroxide solution, increasing the network density of the cryogel and resulting in a slow relaxation of the network chains, with reduced swelling and elasticity [44]. It is worth noting that the pH levels of the gel precursor during and after complete gelatinization with 0.32% WL, 0.32% RL, and 0.16% Ca(OH)_2_ were similar (as seen in the Supplementary Materials, Figure S3), and this may influence the oxidation of the hydroxyl groups of starch, leading to the cryogels have similar properties despite the different concentrations of Ca^2+^ in the solutions.

**Table 1:** Porosity and swelling ratio of the cryogels (*n* = 3).

Type of Cross-Linker	Concentration of Cross-Linker (%*w/v*) ***	Concentration of Ca^2+^ (mg/L) *	Recovers from Compression **	Porosity (%)	Swelling Ratio (S_g/g_)
WL	0.08	117 ± 5	No	-	-
WL	0.16	114 ± 1	No	-	-
WL	0.24	112 ± 0	No	-	-
WL	0.32	119 ± 1	Yes	51 ± 4	5.9 ± 0.3
RL	0.08	176 ± 0	No	-	-
RL	0.16	145 ± 5	Yes	19 ± 2	3.3 ± 0.3
RL	0.24	403 ± 12	Yes	33 ± 3	4.2 ± 0.4
RL	0.32	630 ± 3	Yes	52 ± 6	6.0 ± 0.3
Ca(OH)_2_	0.08	364 ± 5	Yes	39 ± 2	4.5 ± 0.3
Ca(OH)_2_	0.16	419 ± 8	Yes	51 ± 2	6.4 ± 0.6
Ca(OH)_2_	0.24	688 ± 22	Yes	32 ± 8	4.1 ± 0.6
Ca(OH)_2_	0.32	712 ± 8	Yes	28 ± 1	3.6 ± 0.3

*: Analyzed by ICP-OES; **: while squeezing the swollen gel to investigate its porosity; ***: %*w/v* = g of lime or Ca(OH)_2_ in 100 mL of solution.

Therefore, the results showed that limewater and calcium hydroxide solution used as the cross-linker affected the cryogels and the limewater from 0.32% RL and 0.16% Ca(OH)_2_ prepared at least three days ahead of use is recommended as it provided cryogels with good physical and mechanical properties and with the highest porosity. The limewater (60 mL) was used to disperse rice flour (4, 8, 12, and 16 g) to prepare the gel precursor. The cryogels prepared from 4 g of starch were damaged after applying compression, while 8 g starch cryogels could tolerate the compression, indicating greater strength; this may have been due to the increased degree of cross-linking, resulting in smaller macropores in cryogels with higher porosity and better elasticity. However, further increasing rice flour to 12 and 16 g made the cryogels dense and they were damaged after applying compression. Leftover starch granules were also evidenced in the cryogels, which may indicate lack of sufficient water to swell the starch granules and access their internal structures for solubilization [45].

**Figure 3:**
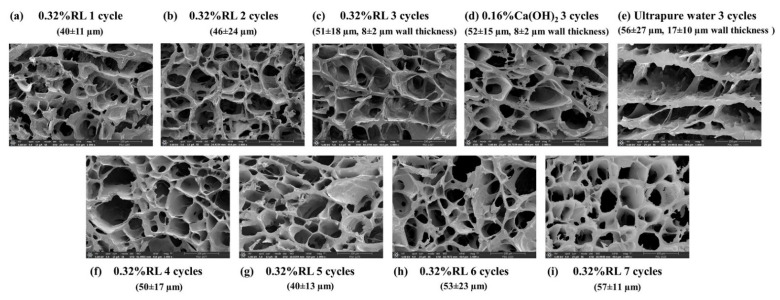
FE-SEM images and average pore sizes of the cryogels prepared by using (**a**–**c**,**f**–**i**) 0.32% RL with 1–7 freeze–thaw cycles, respectively (**d**) 0.16% Ca(OH)_2_, and (**e**) ultrapure water with three freeze–thaw cycles under the optimal conditions.

The influence of freeze thaw cycles on the cryogels was investigated, and the results are shown in the Supplementary Materials (Figure S6). When the number of freeze thaw cycles was increased, the pores of the cryogels seemed to grow from 1 to 3 cycles (40 ± 11 to 51 ± 18 µm; Figure 3a–c) and slightly grow after that (50 ± 17 to 57 ± 11 µm; Figure 3f–i). The cryogenic cycles also affected the porosity, which increased from 1 to 3 cycles and then remained constant from 4 to 7 cycles (Supplementary Materials, Figure S7). When compression was applied to the cryogels, no damage was observed to the material with three cycles, while those from one and two cycles were damaged. This corresponds to the results for noncovalent PVA cryogel, the strength of which increased with increasing the number of freeze thaw cycles, due to the growth of crystallinity in the PVA [46].

Thus, an alternative procedure to prepare a monolithic macroporous cryogel from starch is reported as follows: Rice flour (8 g) was dispersed in 60 mL of limewater from 0.32% RL or 0.16% Ca(OH)_2_, prepared at least 3 days ahead of use. The gel precursor was gradually gelatinized before cooling down, and was tightly filled into a container, and then frozen at −18 °C for 24 h. The resultant monolith is then thawed at room temperature, and freeze thaw treatment was repeated for a total of three cycles. In this manner, a cryogel from starch can be prepared without the addition of any synthetic polymer.

The obtained single-rod cryogels were cut to appropriate sizes depending on their application before drying. When the cryogel was incubated without soaking in ethanol, it lost its shape (Supplementary Materials, Figure S8a, in the middle) due to pore collapse, which commonly occurs in porous materials [34]. The protection against pore collapse by ethanol dehydration may contribute to some degree of crystallization of starch, and hardened surfaces were creased during dehydration [10]. The FE-SEM images of the ethanol-dehydrated cryogel (Figure 3c,d) show thicker walls than in freeze-dried cryogel (Supplementary Materials, Figure S9) as the growth of ice crystals during the freeze-drying process could push aside the pore walls of the materials [34]. The dehydration time, incubation temperature, and incubation time are discussed in the Supplementary Materials (Figure S10). The porosity of the ethanol-dehydrated cryogels was higher than that of the freeze-dried cryogels (Supplementary Materials, Figure S11). The dried cryogels can be stored for at least 2 years, and can be re-conditioned by soaking in water for 10 min before use (Supplementary Materials, Figure S8b,c), i.e., the filters prepared on March 2019 can reduce the light absorption of the water sample (no. 5) at 290 nm with −6.25% difference for 0.32%RL and −7.29% for 0.16% Ca(OH)_2_ prepared in July, 2021. Thus, the cryogels prepared via the novel procedure reported in this work can be dried using a simple and easy process, i.e., ethanol dehydration coupled with incubation in oven for an appropriate time, instead of freeze drying.

**Figure 4:**
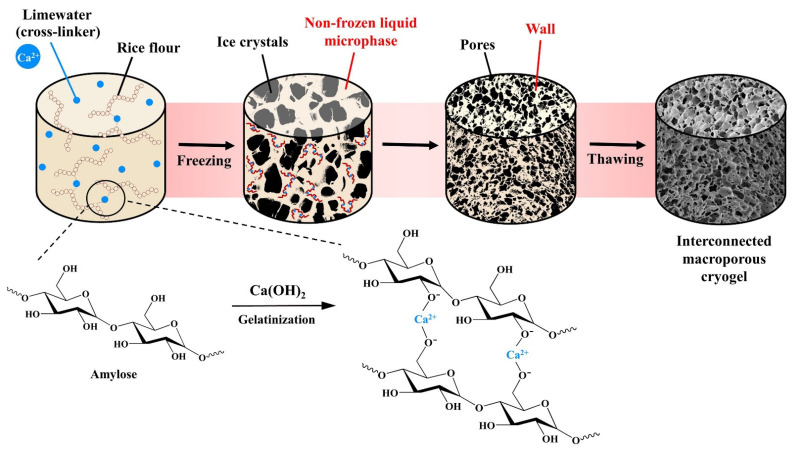
Proposed mechanism of synthesis of the cryogels.

The proposed mechanism is shown in Figure 4, and the FE-SEM images of the cryogels obtained under the optimal conditions are shown in Figure 3c,d. The pore sizes of the cryogels prepared from 0.32% RL (~51 ± 18 µm) and 0.16% Ca(OH)_2_ (~52 ± 15 µm) are smaller than those of cryogels synthesized without the cross-linker (~56 ± 27 µm; Figure 3e), which were larger than those frozen in liquid nitrogen (−196 °C, 2.5 µm), but smaller than the macropores (430.93 µm) formed in the freezer (−15 °C) [47]. The thickness of the walls of the cryogels synthesized without the cross-linker (~17 ± 10 µm) was also the greatest (~8 ± 2 µm for 0.32% RL and 0.16% Ca(OH)_2_); due to reduced interconnected polymerization of the network.

### 3.2. Characterization of Cryogels

**Figure 5:**
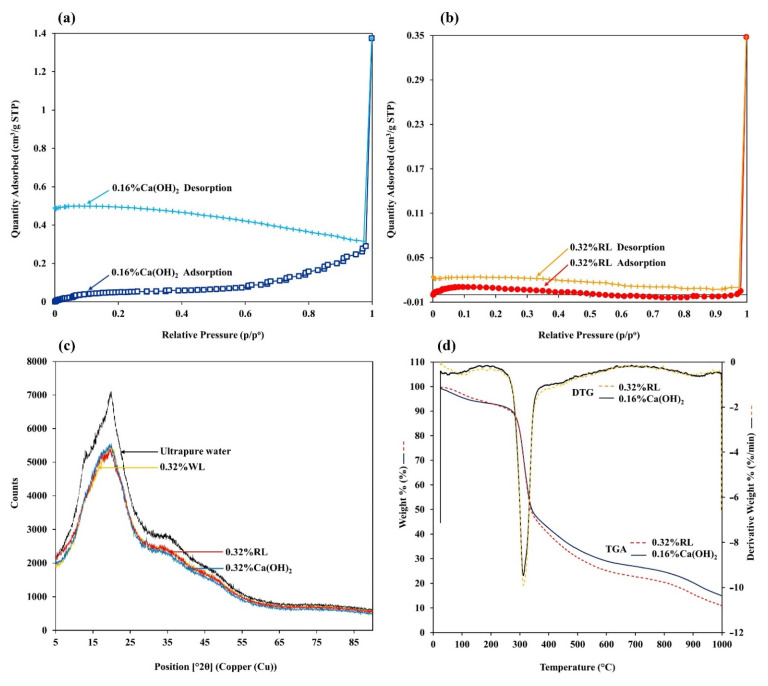
Nitrogen adsorption-desorption isotherms of the cryogels prepared from (**a**) 0.16% Ca(OH)_2_, and (**b**) 0.32% RL; (**c**) XRD and, (**d**) TGA patterns of the synthesized cryogels.

The adsorption-based average pore diameter from the BET analysis of 0.32% RL (5.6206 Å) was larger than that of 0.16% Ca(OH)_2_ (3.4121 Å), with less specific surface area (S_BET_) at 1.1177 ± 0.0320 m^2^g^−1^ (4.3117 ± 0.1017 m^2^g^−1^ for 0.16% Ca(OH)_2_). The S_BET_ matches other reports on a low surface area (3 to 13 [34] and 0.6 to 7.7 m^2^g^−1^ [48]) for porous materials synthesized from native starch using freeze-drying. These results indicate that 0.32% RL and 0.16% Ca(OH)_2_ seem to be macroporous rather than meso- or microporous materials, matching the FE-SEM images showing ~50 µm pore size. Both prepared cryogels exhibited a typical type III adsorption isotherm (Figure 5a,b), as there was no steep uptake at low P/P_0_ indicating that the N_2_ was adsorbed on the surface of the macroporous materials, with relatively weak interaction [49]. Opened loops were also observed for both cryogels, which could be attributed to several factors. Since cryogels prepared from starch do not have a rigid structure, they can deform (swell) during adsorption or pore filling. The trapped nitrogen also cannot be released due to the affinity of nitrogen in cryogels caused by the heterogeneity of the cryogel surface [50].

The XRD patterns of the cryogels prepared with and without Ca^2+^ revealed a broad hump centered at 2θ ~ 20° (Figure 5c), indicating an amorphous structure. The intense XRD peaks typically present at 2θ values of 12.2°, 15.1°, 17°, 18.1°, 20°, 23.1°, and 26.6° indicate an A-type crystalline structure of rice starch [51], but these were not observed. This is because the crystallinity of the starch granules was disrupted by gelatinization at an elevated temperature [52], as well as the cross-linking with Ca^2+^. It can be seen that the cryogel prepared from ultrapure water showed small peaks located at 2θ ~ 13° and 2θ ~ 20°, which are characteristic of a V-type crystalline structure [39,51]. This indicates that some crystallinity remained in the cryogel prepared without Ca^2+^, while the intensity of these peaks was much reduced in the cryogels with Ca^2+^. These results suggest that Ca^2+^ ions also contribute to the loss of crystallinity.

Thermal gravimetric analysis (TGA) of the cryogels prepared from 0.16% Ca(OH)_2_ and 0.32% RL showed similar patterns, exhibiting four main steps from room temperature to 1000 °C (Figure 5d): The ~10% weight loss from room temperature to 200 °C was observed at the first step due to loss of moisture and/or other low-molecular weight compounds [39]. The second step was the major weight loss from 250 to 400 °C, attributed to starch decomposition corresponding to hydroxyl group elimination and carbon chain decomposition and depolymerization [39]. The third step occurred at 400 to 600 °C attributed to Ca(OH)_2_ dehydroxylation, while the fourth step occurred above 600 °C indicating the loss of chemically bound CO_2_ (decarboxylation) from CaCO_3_ components [39]. Although the thermal stability of the cryogels prepared with 0.16% Ca(OH)_2_ and 0.32% RL was similar (with maximum degradation rates at 312.1 °C and 312.6 °C, respectively), the weight loss in the third and the fourth steps of the cryogels prepared with 0.32% RL was higher than with 0.16% Ca(OH)_2_ and this may be attributed to the higher amount of Ca(OH)_2_ and chemically bound CO_2_ from CaCO_3_ components.

**Figure 6:**
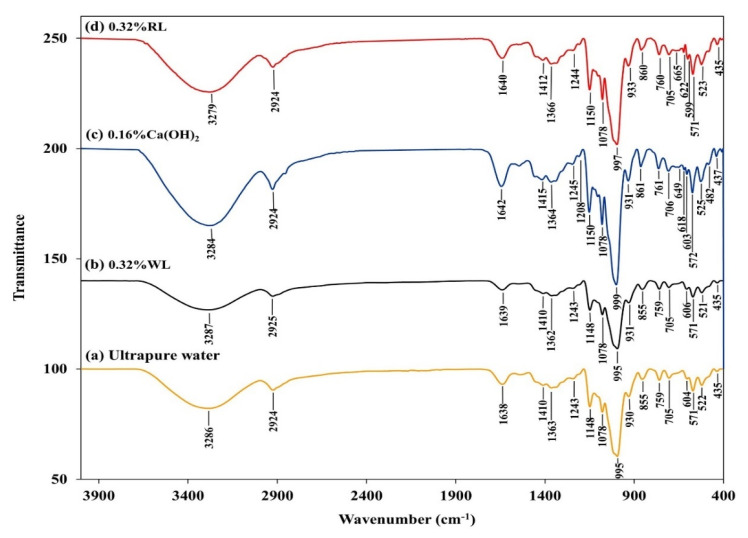
FTIR spectra of the cryogels.

All of the cryogels had similar FTIR spectra (Figure 6) and the major bands are summarized in the Supplementary Materials (Table S12). A large band observed at 3286 cm^−1^ in the cryogels prepared using ultrapure water slightly shifted to a lower absorption frequency of 3284 cm^−1^ and 3279 cm^−1^ in the cryogels prepared with 0.16% Ca(OH)_2_ and 0.32% RL, respectively, indicating stronger interactions of Ca^2+^ with the –OH groups of starch molecules [9,53,54], with a higher concentration of Ca^2+^ in the solution (Table 1). Absorption peaks at 2924 cm^−1^ and 1078 cm^−1^ were consistent for all cryogels, indicating that the chemical bonds did not suffer any changes. The adsorption peaks at 1638, 1410, 1148, 995, and 930 cm^−1^ in the cryogels without Ca^2+^ revealed slight shifts to higher adsorption frequencies of 1642, 1415, 1150, 999, and 933 cm^−1^, respectively, in the cryogels with Ca^2+^. These shifts indicate interactions between the Ca^2+^ and hydroxy groups of starch molecules that can stabilize these bonds, and may contribute to decreased hydrogen bonding. From the results, since no new chemical bonds were observed in the FTIR spectra of the cryogels prepared with Ca^2+^, the divalent ions would interact with starch molecules by physical cross-linking via Van der Waals interactions, consistent with previous reports [42].

### 3.3. Water Uptake Capacity, Swelling Capacity, and Water Retention of Cryogels

**Figure 7:**
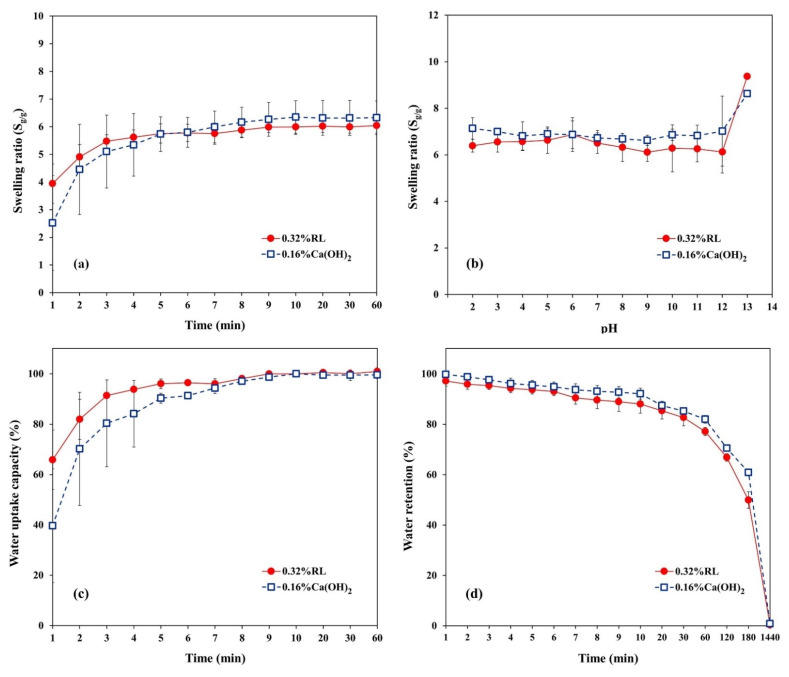
Swelling behavior of the cryogels prepared using 0.32% RL and 0.16% Ca(OH)_2_ (*n* = 3) (**a**) swelling ratio, (**b**) effects of pH, (**c**) water uptake capacity, and (**d**) water retention.

The swelling ratios of the cryogels increased over time at room temperature (Figure 7a). Although the swelling ratio of the 0.16% Ca(OH)_2_ cryogel was less than for 0.32% RL for 1–5 min, the two ratios were similar after reaching the equilibrium at 10 min (6.4 ± 0.6 g_water_/g_cryogel_ for 0.16% Ca(OH)_2_ and 6.0 ± 0.3 g_water_/g_cryogel_ for 0.32% RL). Both cryogels had swelling capacity similar to that in a previous report, namely, 3.2 to 5.5 g/g for polyacrylamide cryogel [11], which is much less than for a conventional gel prepared at ambient temperature. It has been reported that the swelling degree of cryogels is less than that of hydrogels prepared under similar conditions except for the higher temperatures, by at least 3–6-fold [55], or as much as 930-fold [15]. The low swelling ratio of the prepared cryogels makes them suitable for application as filters.

The swelling ratio of both cryogels seemed to be constant for a wide pH range, but not under strongly alkaline conditions (pH 13) (Figure 7b). The pH-independent swelling of the cryogels might relate to the physical cross-linking with Ca^2+^ ions around the hydroxyl groups in the cryogel network, which may limit the additional cross-linking from ions in the solution. The dramatic increase in swelling ratio at high pH may be attributed to the loss of the physical cross-linking between Ca^2+^ ions and the hydroxyl groups in the cryogel network. The results indicate that the material could be applied in a wide pH range without changes in swelling properties. The swelling ratio of both cryogels increased with temperatures (as discussed in the Supplementary Materials (Figure S13) consistent with previous reports [10,56].

The cryogels prepared from 0.32% RL could adsorb water for 599 ± 27% of the dry weight, while 635 ± 59% was recorded for the 0.16% Ca(OH)_2_ cryogel after 10 min (Figure 7c). The water retention abilities of both cryogels decreased over time (Figure 7d). The 0.32% RL cryogel lost 50% of adsorbed water in 3 h, more than the 0.16% Ca(OH)_2_ cryogel which lost 39%. Moreover, the latter showed higher water retention than the former. As discussed in connection with the FTIR results, the interaction between the Ca^2+^ and –OH groups of starch molecules in the 0.32% RL cryogel was stronger than 0.16% Ca(OH)_2_ cryogel, due to the higher concentration of Ca^2+^. The hydrogen bonding between water and the hydroxyl groups which delayed the evaporation of water in the 0.32% RL cryogel was thus less than in the 0.16% Ca(OH)_2_ cryogel, causing the lower water retention.

### 3.4. Swelling Kinetics of Cryogels

The swelling kinetics of the cryogels were investigated and are discussed in the Supplementary Materials (Figure S10). The theoretical equilibrium swelling ratio (Seq) for the 0.16% Ca(OH)_2_ cryogel was higher than that for the 0.32% RL cryogel, with a lower swelling rate constant (ks) and lower initial swelling rate (r_i_) (Supplementary Materials, Table S15). These were close to the experimental swelling ratios at equilibrium at 10 min. The higher initial swelling rate of 0.32% RL also matched the experimental results in Figure 7a.

The swelling mechanism of the cryogels was also determined and is discussed in the Supplementary Materials (Figures S16 and S17). Due to the swelling exponent *n* ≤ 0.5 for both cryogels (Supplementary Materials, Table S15), there was Fickian diffusion with a slower rate of water diffusion than the polymer relaxation rate [44], causing a low swelling capacity for both cryogels. The diffusion coefficient of water through the 0.16% Ca(OH)_2_ cryogel was higher than for the 0.32% RL cryogel, indicating faster diffusion of water in 0.16% Ca(OH)_2_ leading to a greater swelling ratio.

### 3.5. Application of Cryogels as Filters

**Figure 8:**
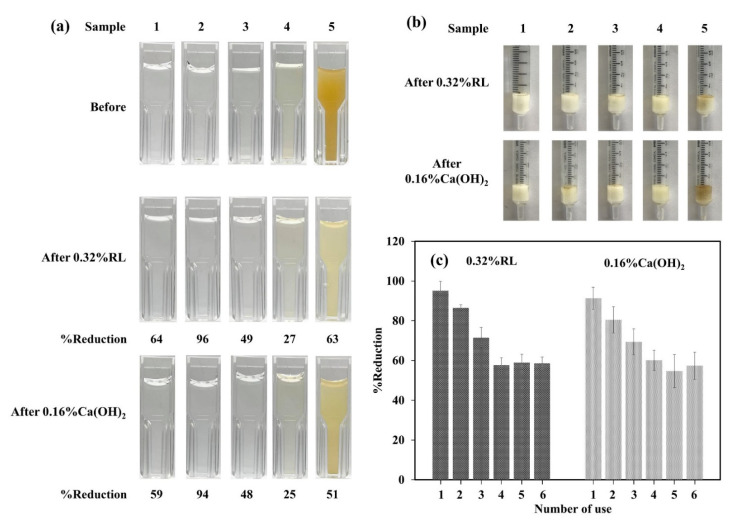
(**a**) Real water samples before and after filtering through the cryogels; (**b**) the cryogels after used to filter real samples; (**c**) reusability of the filters.

Both cryogels were preliminarily tested as filters for five water samples, and showed the ability to reduce the light absorption of the samples at specific wavelengths (Table 2 and Supplementary Materials, Figure S18). The maximum adsorption at ~285–290 nm of these samples decreased from 25 ± 3 to 94 ± 6% and from 27 ± 4 to 96 ± 5% when filtered through the filters prepared from 0.16% Ca(OH)_2_ and 0.32% RL, respectively. It can be seen that the suspended particles in the samples were filtered off (Figure 8a,b). The water samples could also be easily filtered using gravity flow without any need for a vacuum pump, as the prepared filters contained interconnected macropores. In addition, they could be easily cleaned by flushing with reversed flow and washing with ultrapure water, and could be re-used up to three times with ~24% difference to the first use (Figure 8c). These filters cost only THB 0.01 (USD 0.0004) per item as tested, and pose no risk to the environment, so they can also safely be discarded after a single use. These results indicate that the cryogels could have environmental applications.

### 3.6. Biodegradation of Cryogels

Biodegradation of the cryogels was investigated using a soil burial test and it was found that both cryogels lost 100% of their weight when buried in the soil for 30 days (Supplementary Materials, Figure S19) due to hydrolysis and microorganisms [33].

## 4. Conclusions

The procedure to prepare the traditional dessert “Lod-chong” in conjunction with conventional freeze thaw techniques can be applied as an alternative method to prepare starch-based cryogels. Rice flour was used as the natural starch polymer source without any need of a synthetic polymer, while limewater acted as the cross-linker. The Ca^2+^ ions of the latter physically cross-linked with OH groups in starch via Van der Waals interactions. Macropores evidenced in the prepared cryogels (51 ± 18 to 52 ± 15 µm) could facilitate porous flow in applications as filters and/or adsorbents to remove some particles and substances from water. The low swelling capacity of the cryogels (6.0 ± 0.3 to 6.4 ± 0.6 g_water_/g_cryogel_) could be a benefit when used as filters, as well as in some other applications. The material can be easily cleaned and re-used up to three times, and is completely biodegraded when buried in the soil for 30 days. The cryogels reported in the work are thus an attractive choice as cost-effective green materials prepared via a simple procedure. Other types of environmental applications in the removal of environ-mental pollutants are under investigation, and the results will be reported in the future.

## Figures and Tables

**Table 2:** The absorbance of aqueous suspension samples before and after filtering with the prepared filters.

Sample	Type	Absorbance at λ_MAX_ (290 nm)			%Reduction	
		before	after 0.32%RL	after 0.16%Ca(OH)_2_	0.32%RL	0.16%Ca(OH)_2_
1	Surface water	0.192 ± 0.005	0.069 ± 0.005	0.078 ± 0.008	64 ± 3	59 ± 5
2	Surface water	0.111 ± 0.006	0.004 ± 0.005	0.007 ± 0.006	96 ± 5	94 ± 6
3	Surface water	0.193 ± 0.006	0.098 ± 0.003	0.100 ± 0.002	49 ± 1	48 ± 2
4	Surface water	1.148 ± 0.032	0.836 ± 0.038	0.856 ± 0.021	27 ± 4	25 ± 3
5	Soil extract	0.886 ± 0.129	0.319 ± 0.047	0.430 ± 0.064	63 ± 10	51 ± 9

## Data Availability

All data are available from the corresponding author on reasonable request.

## Supplementary Materials

The following are available online at https://www.mdpi.com/article/10.3390/polym13223975/s1: Figure S1: FTIR spectra of white lime (WL), red lime (RL), and calcium hydroxide standard (Ca(OH)_2_); Figure S2: XRD patterns of red lime (RL), white lime (WL), and calcium hydroxide standard (Ca(OH)_2_); Figure S3: pH of calcium hydroxide solutions before and during mixing with starch while heated, and after stopping heating, with various amounts of red lime (RL), white lime (WL), and calcium hydroxide standard solution (Ca(OH)_2_) (*n* = 3); Figure S4: pH of limewater with various amounts of red lime (RL), white lime (WL), and calcium hydroxide standard solution (Ca(OH)_2_) for 28 days after preparation (*n* = 3); Figure S5: Preparation of cryogels from rice starch (right) and Lod-chong noodles (left); Figure S6: (a) Images of the synthesized cryogels from 0.32% WL, 0.32% RL, and 0.16% Ca(OH)_2_ using 1–7 freeze- thaw cycles; (b) FE-SEM images of 0.32% RL cryogels with 1–7 freeze thaw-cycles; Figure S7: Porosity of the synthesized cryogels using 1–7 freeze- thaw cycles; Figure S8: Images of cryogels prepared using 0.32% RL (a) after thawing (left), after drying without (middle) and with (right) soaking in ethanol; (b) top view of the ethanol dehydrated cryogel (left) and re-swollen cryogel (right); and (c) side view of the ethanol dehydrated cryogel (left) and re-swollen cryogel (right); Figure S9: FE-SEM images and the average wall thicknesses of (a) 0.32% RL and (b) 0.16% Ca(OH)_2_ cryogels when dried via freeze-drying; Figure S10: Weight change of the cryogels before and after soaking in ethanol for various incubation times; Figure S11: Porosity of the cryogels dried via ethanol dehydration and via freeze-drying; Table S12: Major FTIR bands in the cryogels [42,52,53,54]; Figure S13: Effect of temperature on the swelling ratios of cryogels prepared using 0.32% RL and 0.16% Ca(OH)_2_; Figure S14: Relationship between t/S and t from investigation of the swelling kinetics of the cryogels; Table S15: Swelling and diffusion characteristics of the cryogels (*n* = 3); Figure S16: Relationship between ln S_F_ and ln t from investigation of the swelling mechanisms of the cryogels; Figure S17: Diffusion curves of the prepared cryogels; Figure S18: Absorption spectra of water samples before and after filtration using the prepared filters; Figure S19: %Weight remaining of the prepared cryogels after burial in soil for 30 days.
