# Supplementary material for: Preparation and Characterization of Calcium Cross-Linked Starch Monolithic Cryogels and Their Application as Cost-Effective Green Filters
Source: Polymers (Basel). 2021 Nov 17;13(22):3975. doi: 10.3390/polym13223975 (PMC8617897; doi:10.3390/polym13223975)
Supplement: Supplementary file 1 [file polymers-13-03975-s001.zip › polymers-1443238-supplementary.pdf]

Electronic Supplementary Material

Preparation and characterization of calcium cross-linked starch monolithic cryogel and its application as a cost-effective green filter

Chanita Boonkanon [1], Kharittha Phatthanawiwat [1], Laemthong Chuenchom [2], Nareumon Lamthornkit [1], Tarawee Taweekarn [1], Worawit Wongniramaikul [1], and Aree Choodum [1,*]

[1] Integrated Science and Technology Research Center, Faculty of Technology and Environment,
Prince of Songkla University, Phuket Campus, Kathu, Phuket 83120 Thailand; chanitanbkn@gmail.com (C.B.); kharittha.p@gmail.com (K.P.); jar.nareumon@gmail.com (N.L.); T.tarawee@hotmail.com (T.T.); wilasinee.s@phuket.psu.ac.th (W.S.); wadcharawadee.n@phuket.psu.ac.th (W.L.); worawit.won@phuket.psu.ac.th (W.W.)

[2] Division of Physical Science and Center of Excellence for Innovation in Chemistry, Faculty of Science, Prince of Songkla University, Hat Yai Campus, Hat Yai, Songkhla 90110 Thailand; laemthong.c@psu.ac.th (L.C.)

* Correspondence: Email address: aree.c@phuket.psu.ac.th; Tel.: +66(0)7627 6481

Preparation of limewater and its pH

Calcium hydroxide solutions were prepared both in the form of limewater from red lime (RL) and white lime (WL) which is commonly used in the preparation of Lod-chong and standard calcium hydroxide solution from calcium hydroxide standard ($Ca(OH)_2$). It is worth noting that WL is calcium hydroxide that is commonly prepared by adding water to CaO, while RL is the mixture of WL with curcumin. The FTIR spectra and XRD patterns of RL, WL, and $Ca(OH)_2$ are shown in Figure S1 and Figure S2, respectively, indicating that calcium hydroxide is the main component. RL showed similar FTIR and XRD patterns to $Ca(OH)_2$ while WL revealed some differences.

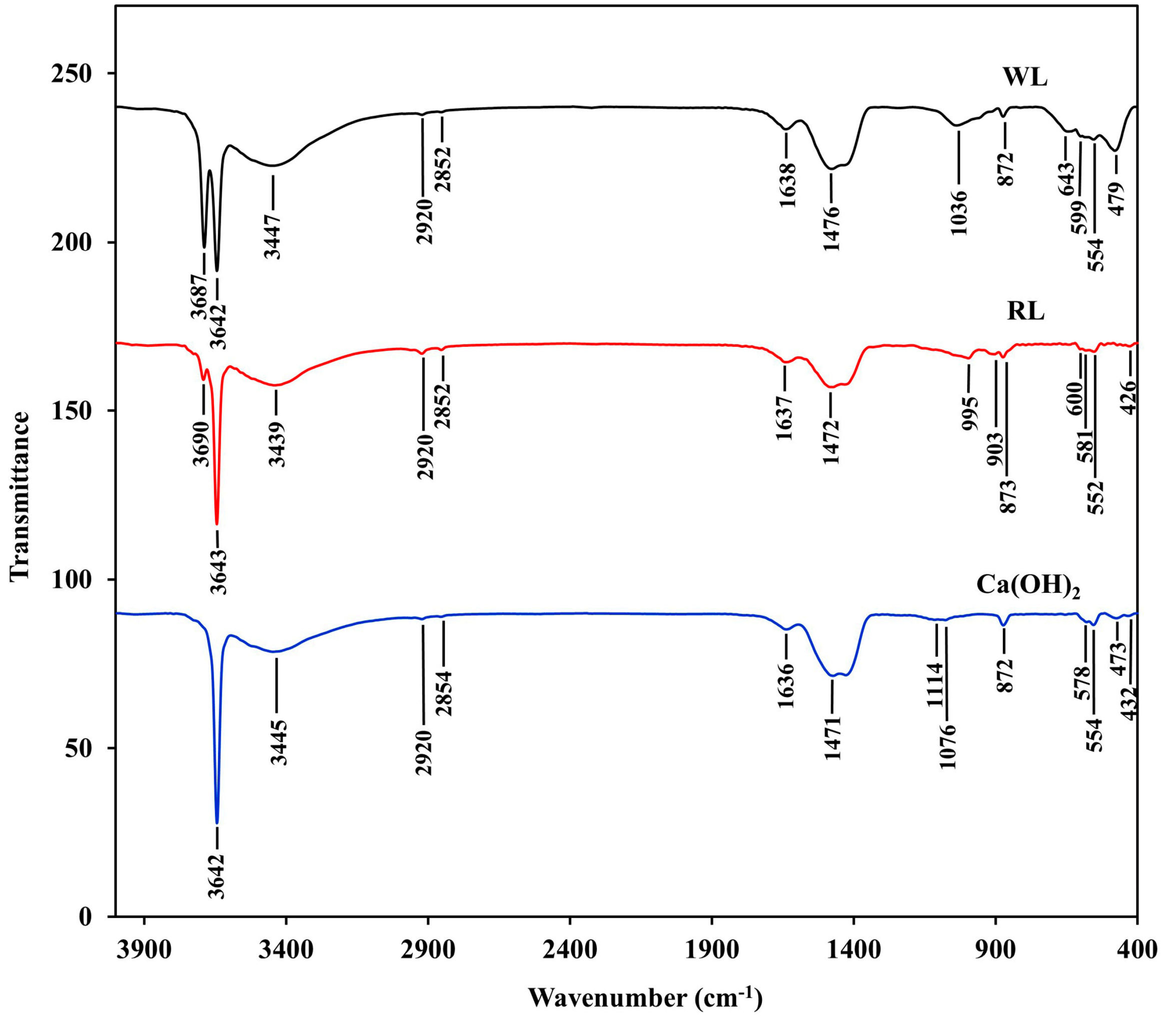

Figure S1. FTIR spectra of white lime (WL), red lime (RL), and calcium hydroxide standard ($Ca(OH)_2$).

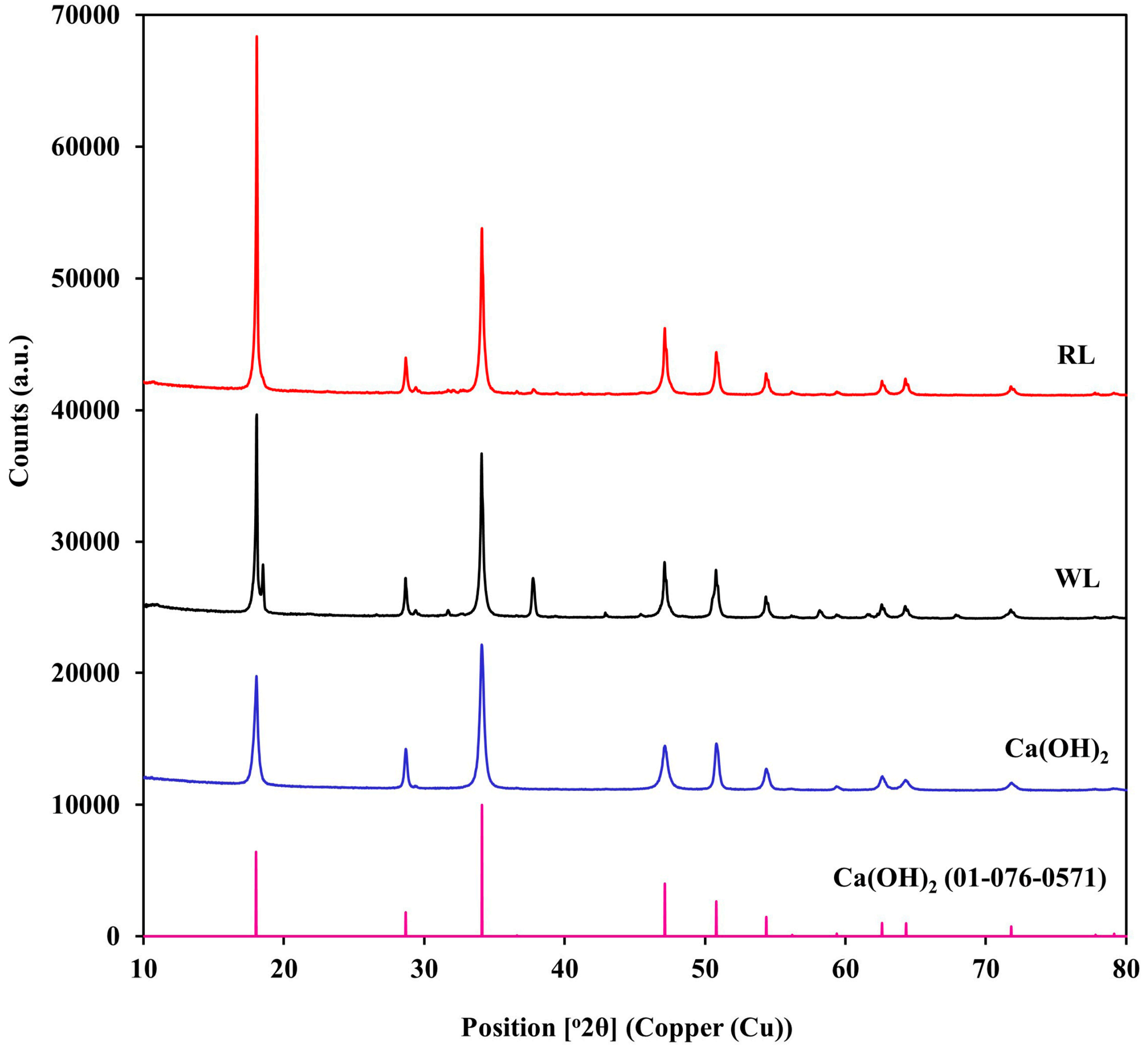

Figure S2. XRD patterns of red lime (RL), white lime (WL), and calcium hydroxide standard ($Ca(OH)_2$).

Various compositions of calcium hydroxide solution were prepared by dissolving 0.08, 0.16, 0.24, and 0.32%w/v of RL, WL, and $Ca(OH)_2$ in ultrapure water. The mixes were left to settle for an appropriate time (1, 3, 5, 7, 14, and 28 days) before taking clear solution to prepare the gel precursor. At the same composition (%), the pH of calcium hydroxide solution prepared from $Ca(OH)_2$ was higher than the pH of limewater prepared with RL and WL. This indicates that RL and WL may contain other components apart from calcium hydroxide reducing the amount of calcium hydroxide below the standard. When the content of calcium hydroxide or lime in the solution was increased from 0.08 to 0.40%, the pH increased with the amount of hydroxide ions, i.e. 12.2 to 12.6 for $Ca(OH)_2$, 11.8 to 12.3 for RL, and 11.6 to 12.0 for WL. The pH of $Ca(OH)_2$ solution from $Ca(OH)_2$ standard slightly increased after 0.16% and became constant after 0.24%, indicating that saturation of the solution occurred at some concentration >0.16% and <0.24%. This is in good agreement with the reported solubility of $Ca(OH)_2$ at 0.185 g/100 mL at 0°C and decreases with increasing temperature to 0.077 g/100 mL at 100°C [57]; as well as with the results from previous work (0.1%

<composition< 0.4%) [55]. The pH of RL and WL became constant after 0.32% indicating that the maximum solubility of lime was in the range from 0.24 to 0.32%.

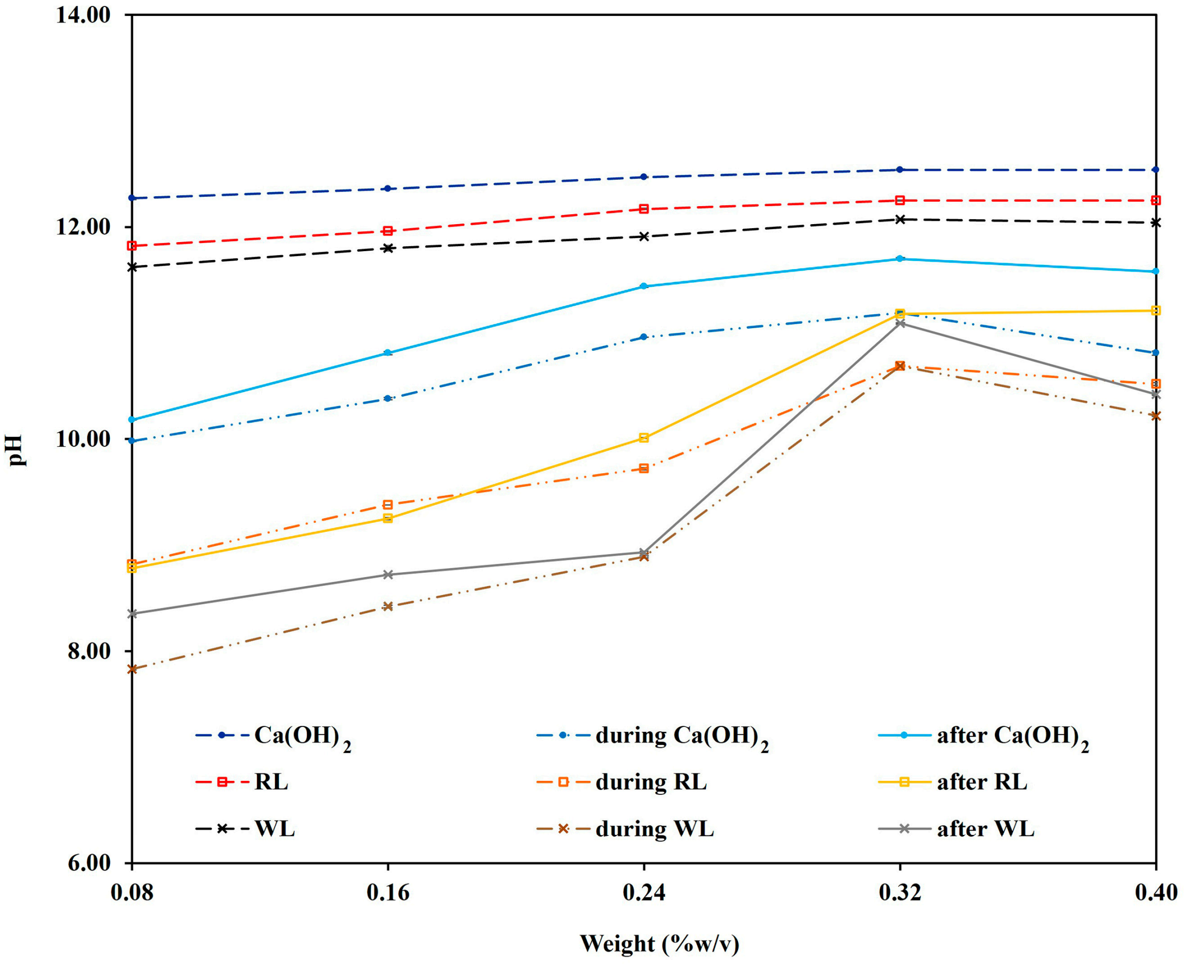

Figure S3. pH of calcium hydroxide solutions before and during mixing with starch while heated, and after stopping heating, with various amounts of red lime (RL), white lime (WL), and calcium hydroxide standard solution ($Ca(OH)_2$) (n =3).

Since the preparation of limewater for making Lod-chong noodles commonly requires 7 days to allow the precipitates to settle leaving a clear solution for use, the pH of the solution was tested for 28 days to confirm the equilibrium. When RL, WL and $Ca(OH)_2$ standard were dissolved in water, turbid solution was firstly obtained, and the pH was found to be in the range from 11.6 to 12.6. The solution was then kept at room temperature allowing precipitation of undissolved particles resulting in a clear solution in an upper layer which would be used to prepare the Lod-chong noodles. The pH of the solution decreased within 2 days of storage, then increased and became constant after 3 days indicating equilibrium. However, it was found that the equilibrium pH was the same as the pH on the day of preparation. Thus, when considering

only pH, the results indicate that the calcium hydroxide solution can be used on the day of preparation by filtering any undissolved particles out or by waiting for at least 3 days before taking clear solution for use.

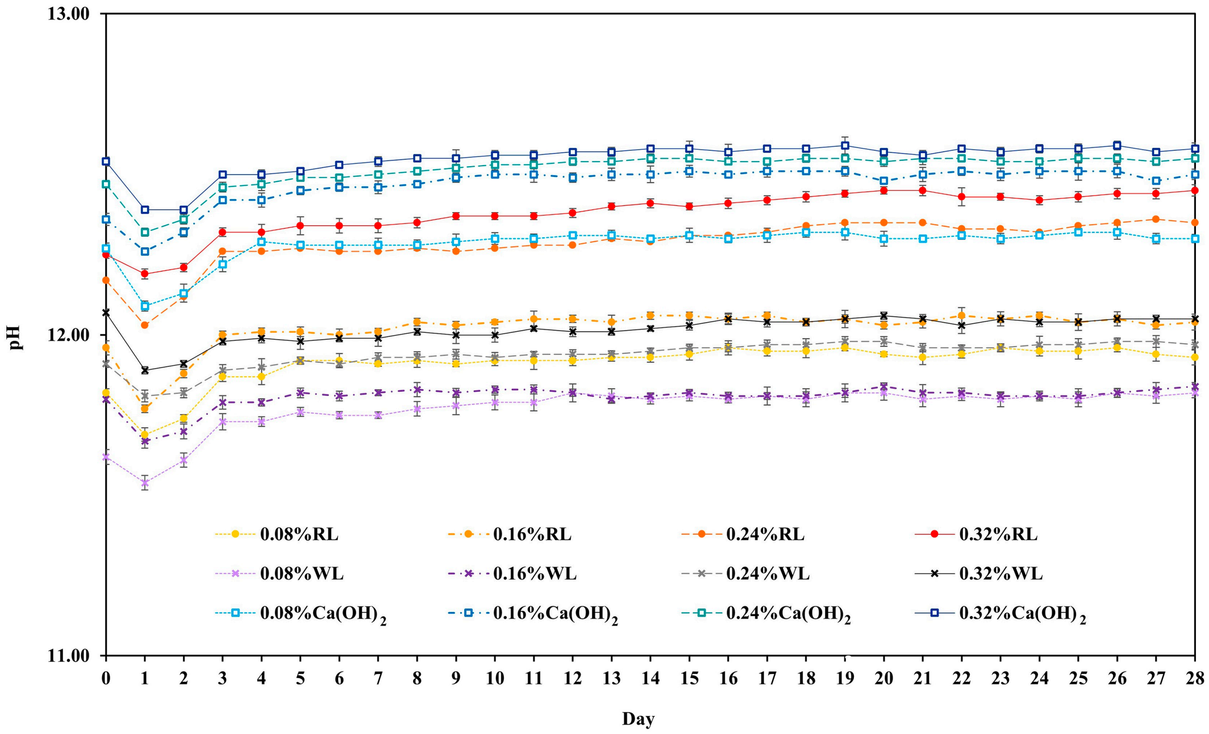

Figure S4. pH of limewater with various amounts of red lime (RL), white lime (WL), and calcium hydroxide standard solution ($Ca(OH)_2$) for 28 days after preparation (n=3).

Preparation of cryogel from starch

Cryogel based on native starch was synthesized by modifying the procedure for preparation of Lod-chong which is the famous Thai dessert. To prepare Lod-chong noodle, rice flour (casava starch, mungbean starch, and arrowroot starch may be added to make noodles look more delicious) is mixed with pandan water prepared by squeezing pandan leaves in limewater and straining. The mixture is cooked with medium heat until the batter starts to set with consistency before turning off the heat. The viscous ingredient is then poured into a Lod-chong press with a bucket below containing cold water to capture the noodles before pressing down in a pumping motion to make the noodles short. This procedure was modified to prepare cryogel as follows. Rice flour (8 g) was dispersed in 60 mL of limewater and gradually heated from 70 to 90°C for 30 minutes until the starch completely gelatinized and became clear. The gelatinized starch was cooled to room temperature under stirring continued for 5 minutes. The homogeneous mixture was tightly filled into 3 mL plastic syringes and then placed in a freezer for freezing at -18°C for 24 hours. The resultant monolith was then thawed at room temperature and subjected to freeze-and-thaw cycles for a specified count. The monolithic cryogel was then removed from the container and cut into smaller pieces (~1 cm in length) to be used as filter.

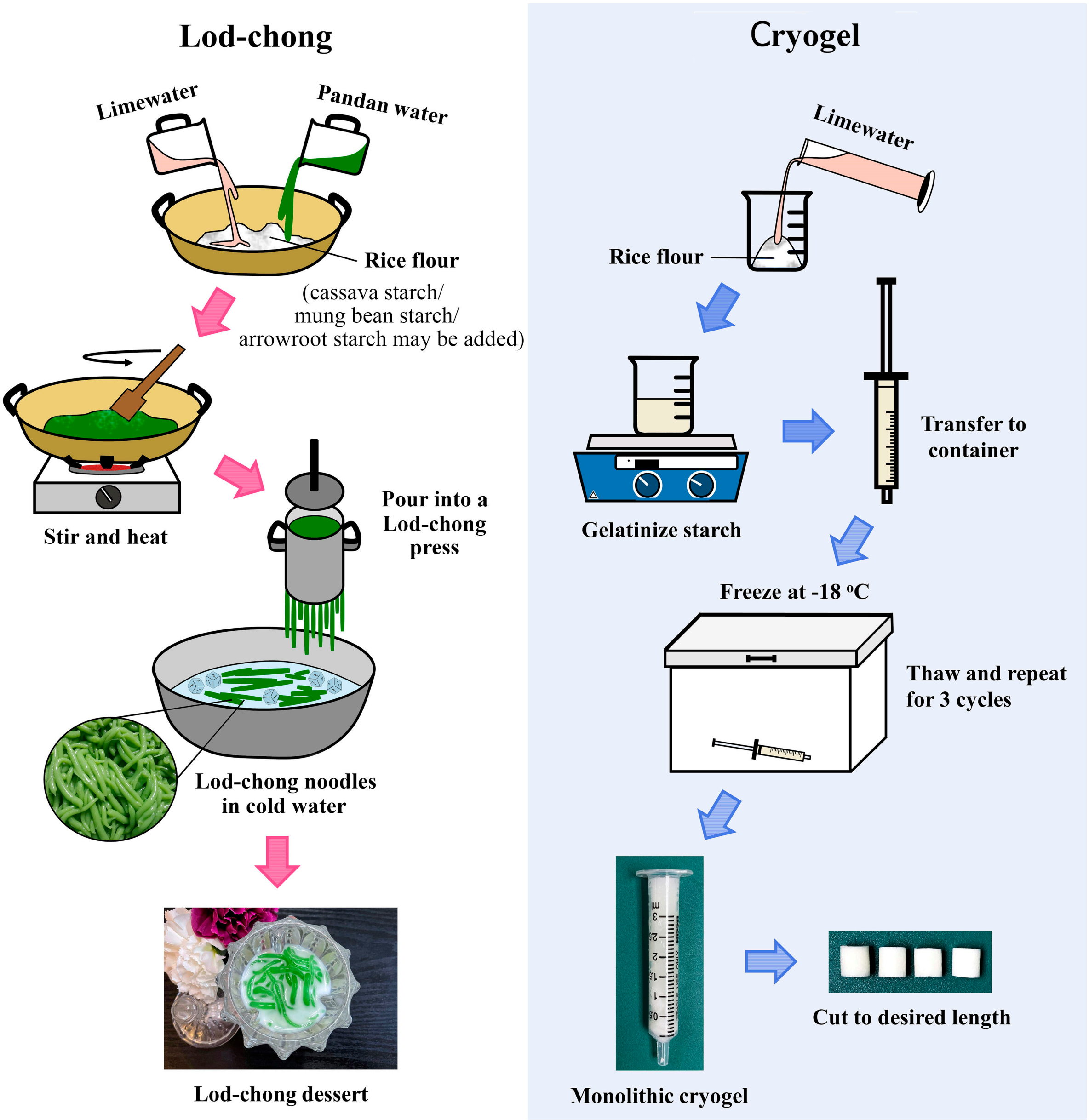

Figure S5. Preparation of cryogels from rice starch (right) and Lod-chong noodles (left).

Influence of freeze and thaw cycles on morphology of the cryogels.

The influence of cryogelation cycles (freeze-and-thaw cycles) was investigated (Figure S6). When freeze-and-thaw cycle count was increased, the pores in the obtained cryogels seemed to grow from 1 to 3 cycles (40±11 to 51±18 μm) and slightly grow after that (50±17 to 57±11 μm) (Fig. 3). When compression was applied to the cryogel, no damage was observed in the material made with 3 cycles, while those from 1 and 2 cycles were damaged. This corresponds to the results for noncovalent cryogel (PVA based cryogel)

whose strength increased on increasing freeze-and-thaw cycles, due to the growth in crystallinity of PVA [46].

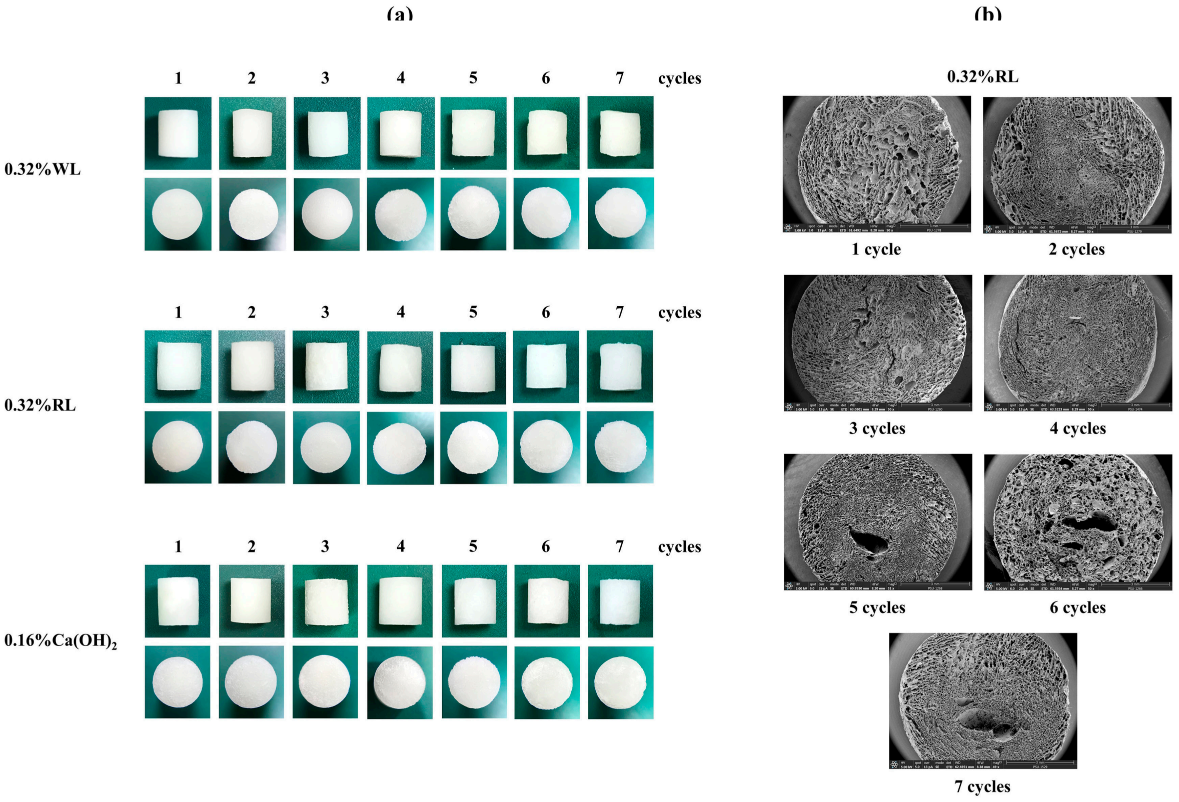

Figure S6. (a) Images of the synthesized cryogels from 0.32% WL, 0.32% RL, and 0.16% $Ca(OH)_2$ using 1-7 freeze- thaw cycles; (b) FE-SEM images of 0.32% RL cryogels with 1-7 freeze– thaw cycles.

Influence of freeze thaw cycles on porosity of the cryogels.

The cryogenic cycles affected the porosity that increased from 1 to 3 cycles and remained constant from 4 to 7 cycles which corresponds to the results for PVA cryogels [46] and a polymer blend of chitosan-PVA [58].

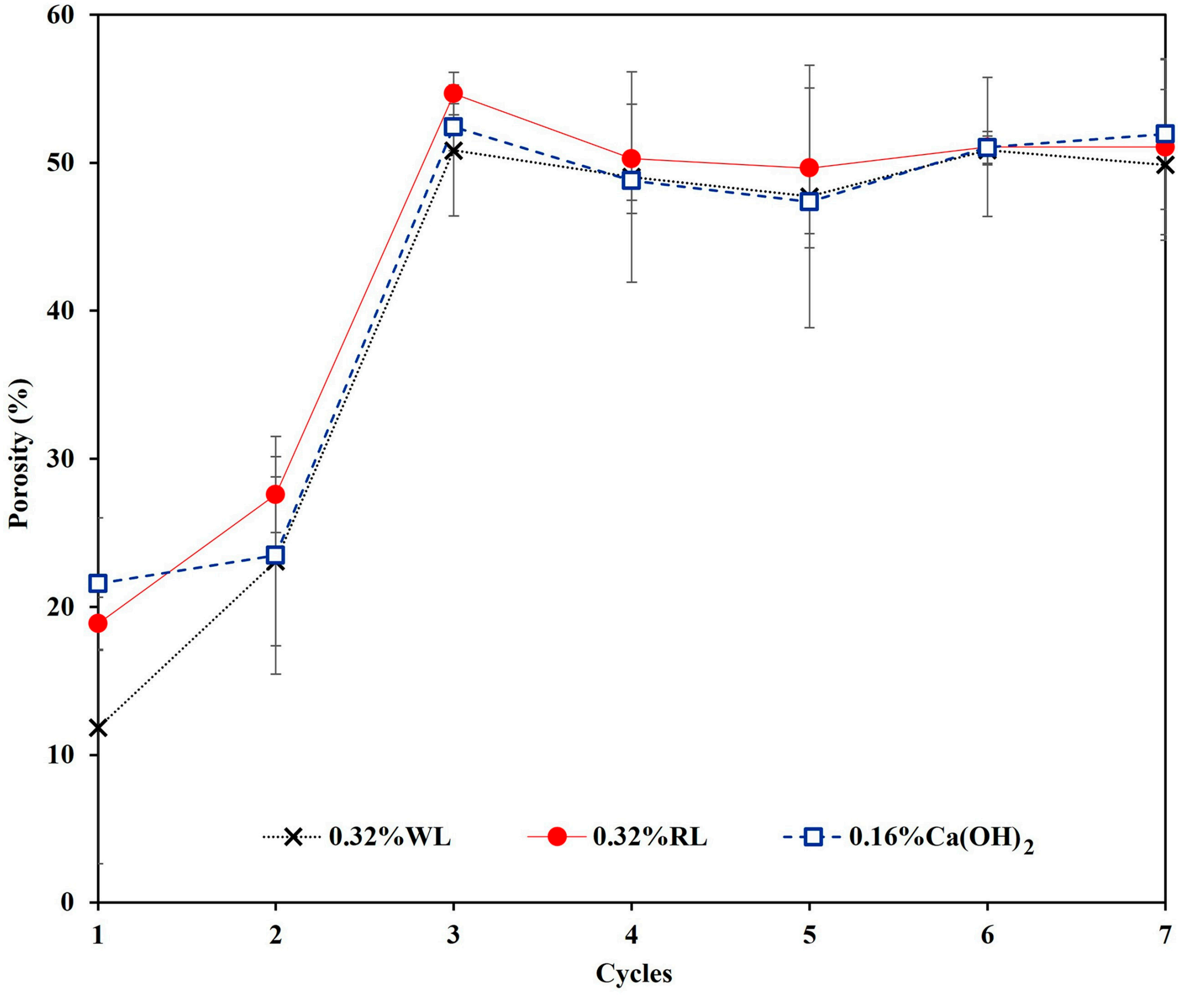

Figure S7. Porosity of the synthesized cryogels using 1- 7 freeze- thaw cycles.

Storage of the cryogels

In order to store the prepared cryogels in ambient conditions prior to use, the single rod cryogels obtained after the freeze-and-thaw process were cut to appropriate size depending on their application, before drying. When the cryogel was incubated without soaking in ethanol, it was out of shape (Figure S8a- in the middle) due to pore collapse during drying that commonly occurs in porous materials [53]. The protection against pore collapse by ethanol dehydration may contribute to some degree of crystallization of starch and hardened surfaces occurred during dehydration [56]. The dried cryogels can be stored for at least 2 years and can be re-conditioned by soaking in water for 10 minutes before use (Figure S8b-c), i.e. the biofilters prepared in March 2019 could reduce the light absorption of the water sample (no. 5) at 290 nm with -6.25% difference for 0.32%RL and -7.29% for 0.16%$Ca(OH)_2$ prepared on July, 2021.

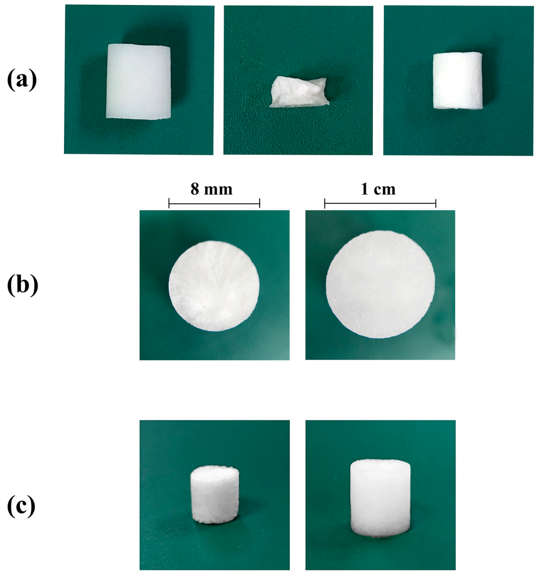

Figure S8. Images of cryogels prepared using 0.32% RL (a) after thawing (left), after drying without (middle) and with (right) soaking in ethanol; (b) top view of the ethanol dehydrated cryogel (left) and re-swollen cryogel (right); and (c) side view of the ethanol dehydrated cryogel (left) and re-swollen cryogel (right).

Influence of drying process on wall thickness of the cryogels.

The FE-SEM images of the ethanol dehydrated cryogel (Fig. 3c-d) show thicker walls than in freeze dried cryogel (Figure S9) as the growth of ice crystals in freeze-drying process could push aside pore walls of the materials [53].

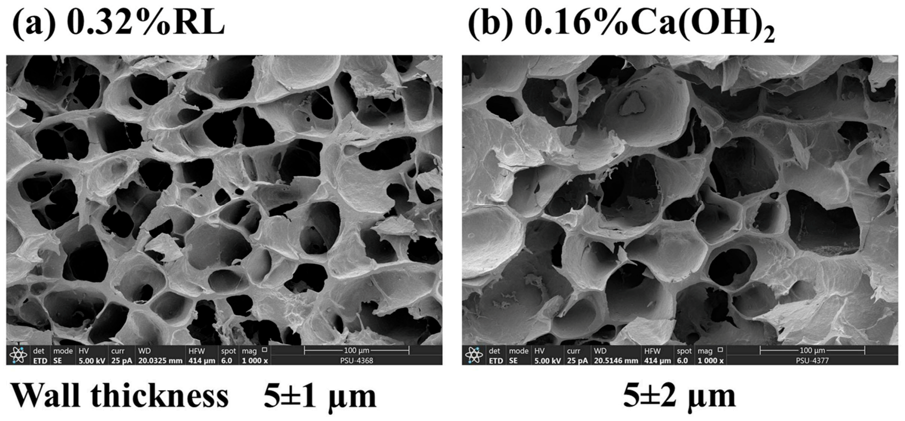

Figure S9. FE-SEM images and the average wall thicknesses of (a) 0.32% RL and (b) 0.16% $Ca(OH)_2$ when dried via freeze-drying.

Optimization of ethanol dehydration time.

Soaking in ethanol before drying the cryogel could protect against pore collapse during drying [53] that commonly occurs in porous material. This may contribute some degree of crystallization of starch and hardening of the surface [56]. The ethanol could also dehydrate the material helping the drying [10] after incubation, which can extend their storage time [59]. It was found that the weight of the cryogel after soaking with ethanol remained constant after soaking for at least 3 hours (Figure S10) indicating complete dehydration. The incubation temperature and time did not affect physical and mechanical properties, thus the dehydrated cryogels were dried at 80°C for 30-60 minutes.

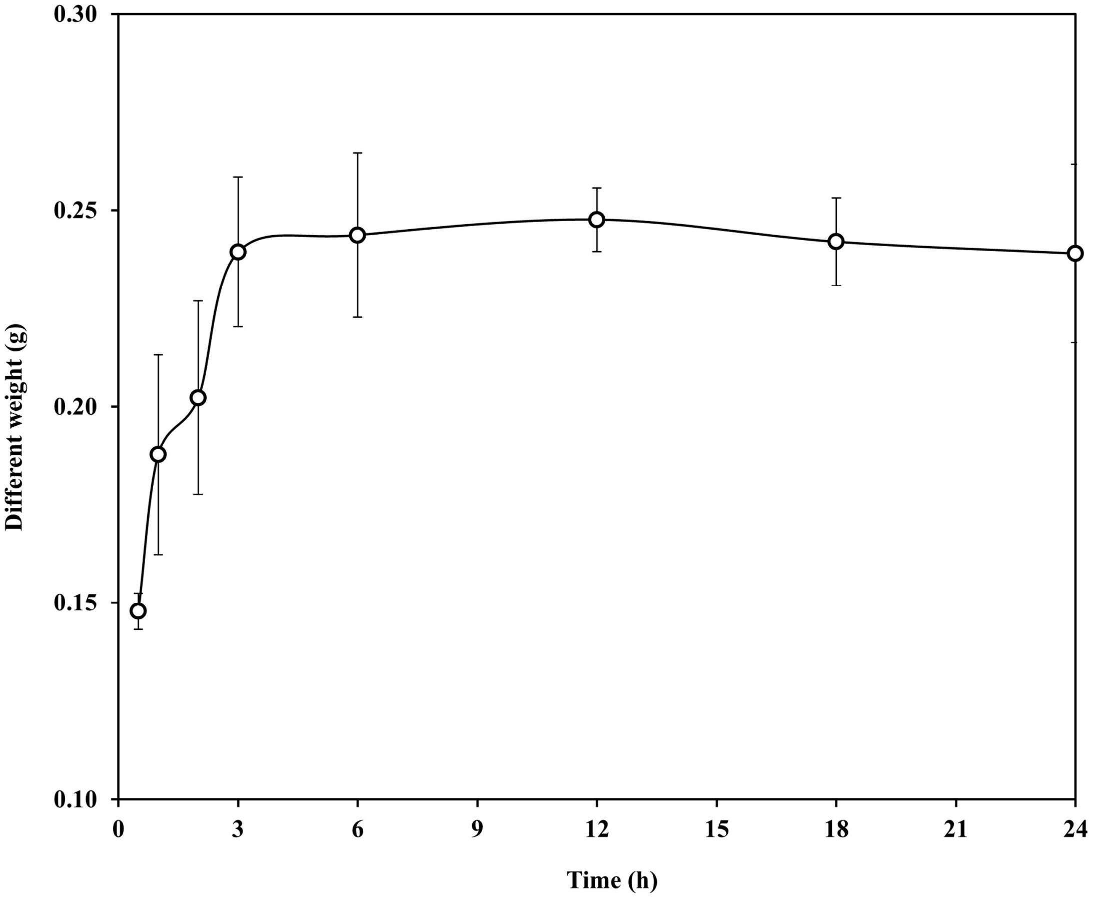

Figure S10. Weight change of the cryogels before and after soaking in ethanol for various incubation times.

Influence of drying process on porosity of the cryogels.

The porosity of the ethanol dehydrated cryogels was higher than those of the freeze-dried cryogels.

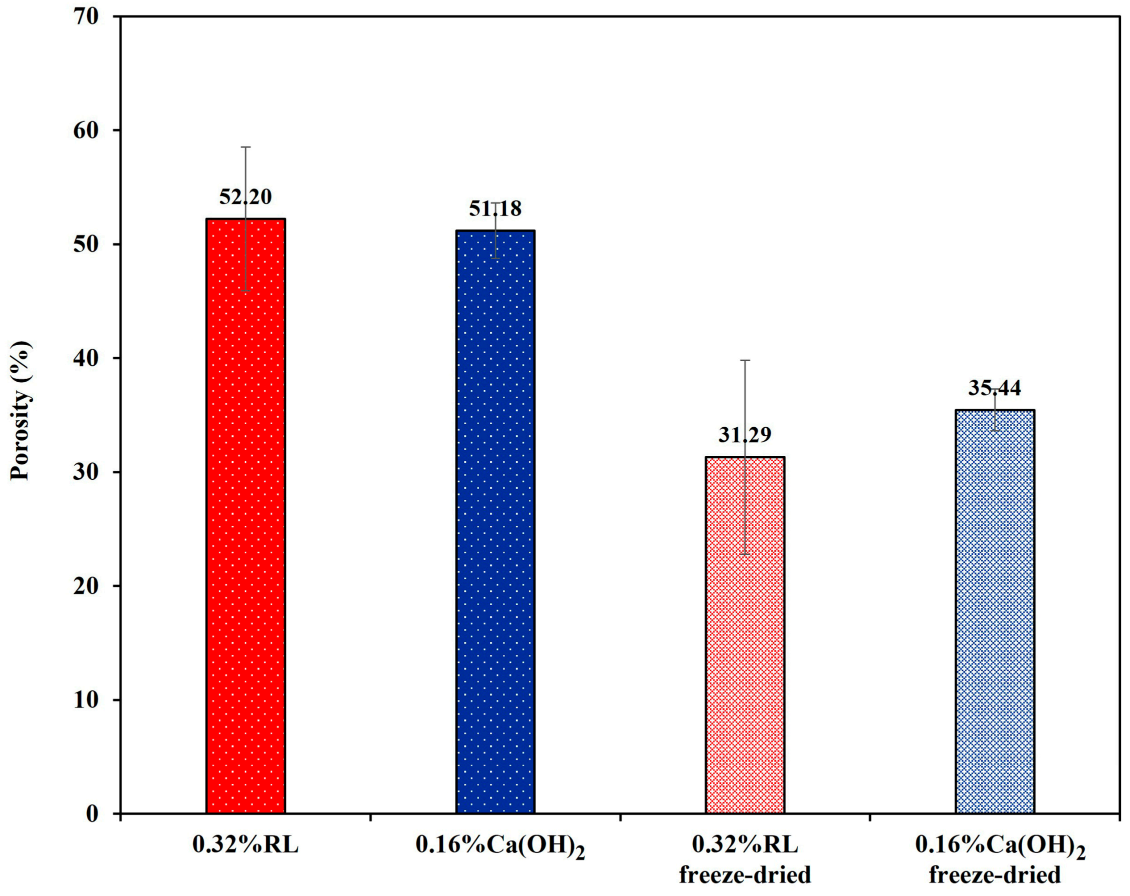

Figure S11. Porosity of the cryogels dried via ethanol dehydration and via freeze-drying.

Table S12. Major FTIR bands in the cryogels [42,52-54]

Band (cm^{-1}) in cryogel prepared from				Vibration mode
**Water**	**0.32%WL**	**0.16%Ca(OH)$_2$**	**0.32%RL**	
3286	3287	3284	3279	O–H stretching (H-bonded)
2924	2925	2924	2924	C–H stretching
1638	1639	1642	1640	Tightly bound water
1410	1410	1415	1412	C–H symmetrical scissoring in CH_2OH
1148	1148	1150	1150	C–O–C asymmetric stretching glycosidic bond
1078	1078	1078	1078	C–O, C–C, O–H bond stretching
995	995	999	997	C–O–H bending
930	931	931	933	C–O–C skeletal of α-glycosidic linkages

Influence of temperature on swelling ratio of the cryogels.

The swelling ratio of both cryogels increased with temperatures from 20 to 60°C similar to the results discussed in literature [55,40]. Swelling is expansion of the cryogel network due to the interaction between the polymeric chains and water molecules, which occurs through entanglement of the interpenetrated polymeric hydrogel network. The swelling ratio thus increases with temperature because the interaction behavior disturbs the disentanglement of interpenetrated polymeric chains and destruction of hydrogen bonding between polymer molecules occurs [56,40].

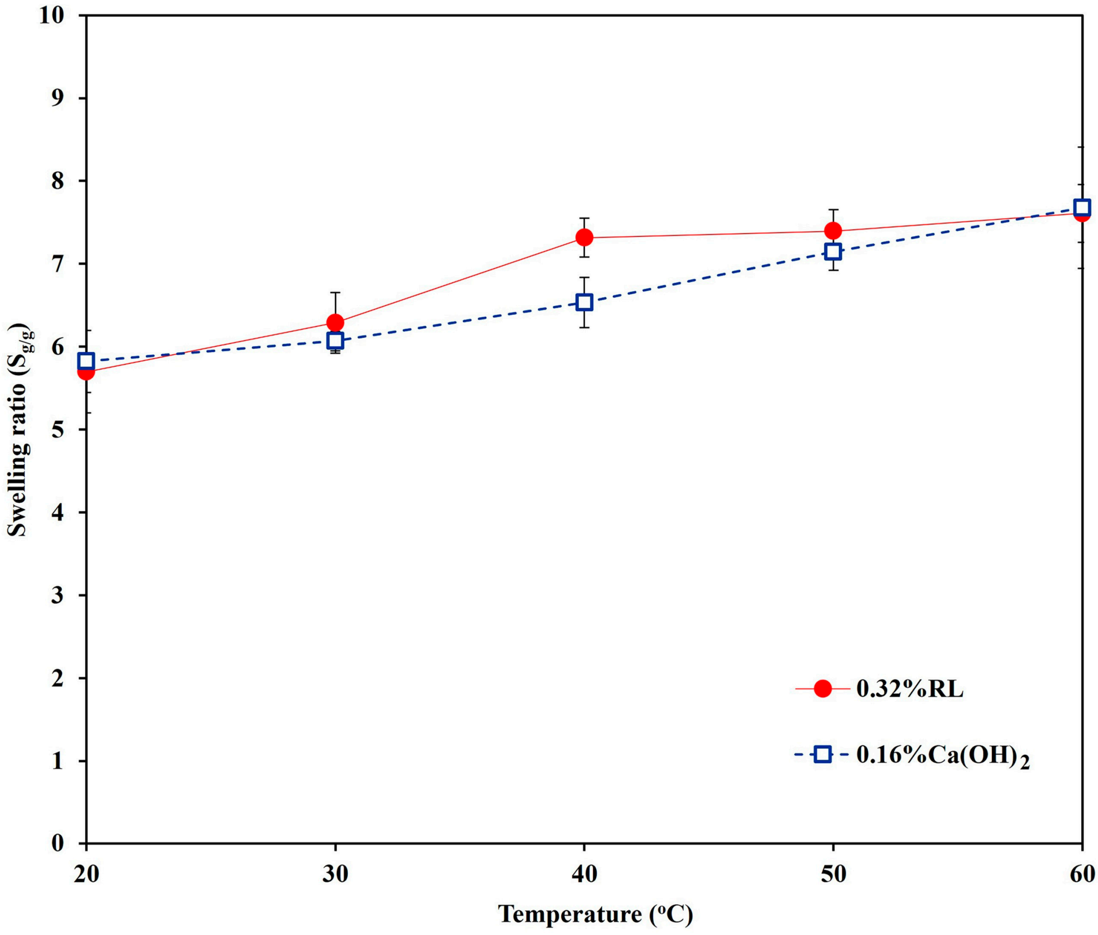

Figure S13. Effect of temperature on the swelling ratios of cryogels prepared using 0.32% RL and 0.16% $Ca(OH)_2$.

Swelling kinetics of the cryogels.

The swelling kinetic mechanism of the prepared cryogel (n=3) was investigated using a second order equation (7) [37,60]:

$$\frac{dS}{dt} = k_s(S_{eq} - S)^2 \quad (7)$$

where dS/dt, k_s, S_{eq}, and S are the rehydration ratio, swelling rate constant, theoretical equilibrium swelling ratio, and swelling ratio at time t, respectively. When definite integration was applied with initial conditions such as $S = 0$ at $t = 0$ and $S = Seq$, equation (8) was obtained [37,60]:

$$\frac{t}{S} = \frac{1}{k_s S_{eq}^2} + \frac{t}{S_{eq}} \quad (8)$$

Thus, the swelling kinetic parameters *i.e.* initial swelling rate (r_i), S_{eq}, and k_s can be investigated by plotting the graph of t/S versus t where $\frac{1}{k_s S_{eq}^2}$ is the intercept that refer to the reciprocal of the initial swelling rate of the material and $\frac{1}{S_{eq}}$ is the slope of the graph (Figure S14). The calculated S_{eq} of the 0.16%$Ca(OH)_2$ (6.4±0.6 g_{water}/g_{crogel}) cryogel was higher than 0.32%RL (6.1±0.3 g_{water}/g_{crogel}) with lower k_s and lower r_i (Table S15). The obtained theoretical equilibrium swelling ratio was close to the experimental swelling ratio at equilibrium at 10 minutes 6.0±0.3 and 6.4±0.6 g_{water}/g_{crogel} for 0.32%RL and 0.16%$Ca(OH)_2$ cryogels, respectively. The higher initial swelling rate (r_i) of 0.32%RL also matched the experimental results in Fig. 7a.

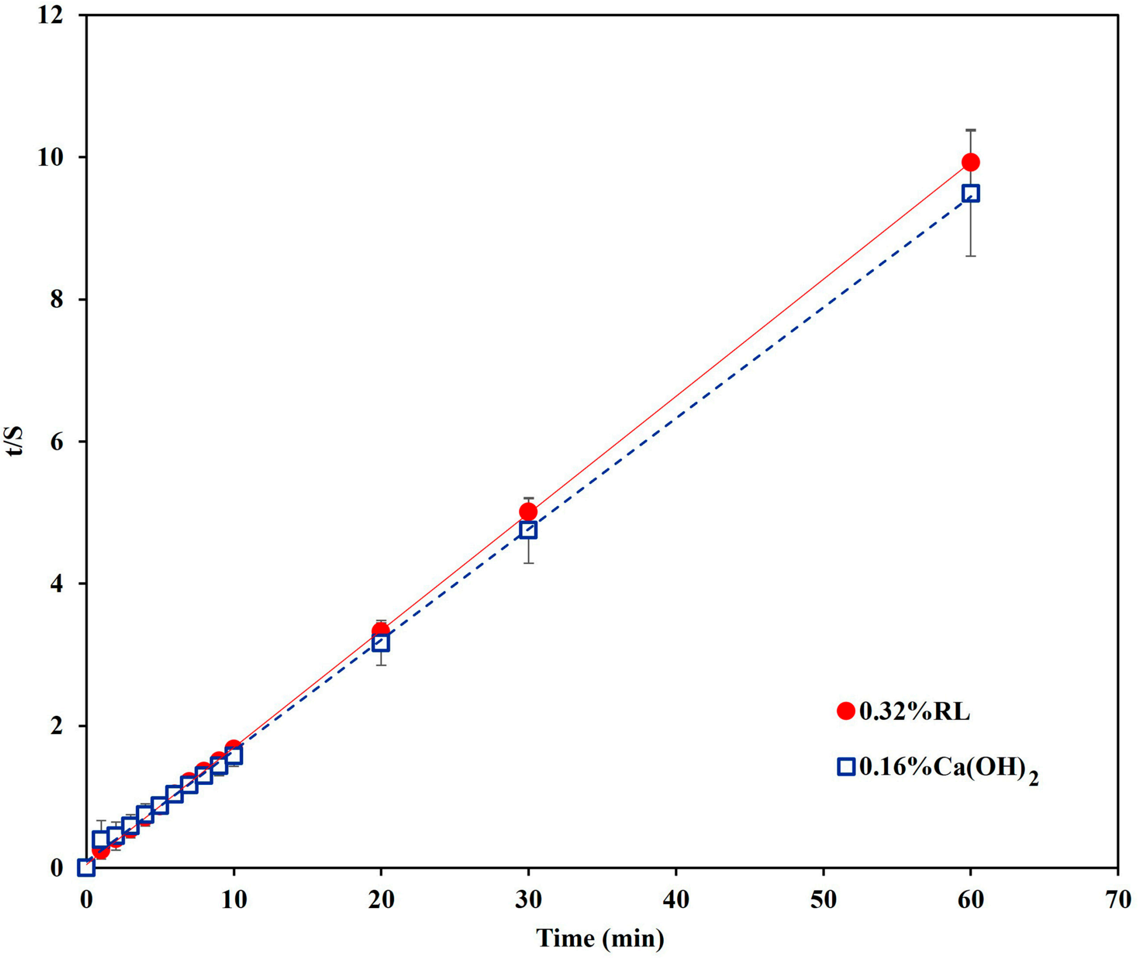

Figure S14. Relationship between t/S and t from investigation of the swelling kinetics of the cryogels.

Table S15. Swelling and diffusion characteristics of the cryogels (n=3).

**Parameters**	**0.16%Ca(OH)₂**	**0.32%RL**
Swelling ratio at equilibrium (S) ($g_{water}/g_{cryogel}$)	6.4±0.6	6.0±0.3
Theoretical swelling ratio at equilibrium (S_{eq}) ($g_{water}/g_{cryogel}$)	6.4±0.6	6.1±0.3
Initial swelling rate (r_i) ($g_{water}/g_{cryogel}$/min)	11±9	21±1
Swelling rate constant (k_s) ($g_{cryogel}/g_{water}$/min)	0.3±0.1	0.57±0.04
Swelling exponent (*n*)	0.2±0.3	0.11±0.05
Diffusion coefficient (cm^2/min)	0.3±0.2	0.1±0.1
%Absorbency	798±39%	836±18%

The swelling mechanism of the cryogels can be determined using the below equation 9 [37,44,60]:

$$S_F = \left(\frac{W_t - W_0}{W_0}\right) = kt^n \quad (9)$$

where S_F is the fraction swelling ratio at time *t*, W_t is the weight of swollen cryogel at the time *t* and W_0 is the weight of dried cryogels at time t = 0, *k* is the swelling constant and *n* is the swelling exponent, which indicates the water transport mechanism [37,44,60]. This equation was used to estimate the *n* value using up to 60% of the swelling ratio values. Thus, when the graph was plotted between ln S_F versus ln *t* (Figure S16), the slope of the obtained straight lines gives the swelling exponent value *n* that could be used to identify the diffusion mechanism [37,44,60]. That is, the diffusion mechanism is a Fickian diffusion when $n \leq 0.5$ (idealize n=0.5 [37,44]), and non-Fickian diffusion or anomalous diffusion when $0.5 < n < 1.0$. If the n=1, the diffusion mechanism is designated as Case II diffusion, and when the n> 1, it is called a super Case II diffusion mechanism, which is very rarely possible [44,52]. Due to the obtained $n \leq 0.5$ (Table S15), the diffusion mechanism of water through both cryogels is Fickian diffusion with a slower rate of water diffusion than the polymer relaxation rate [44] causing the low swelling capacity of both cryogels.

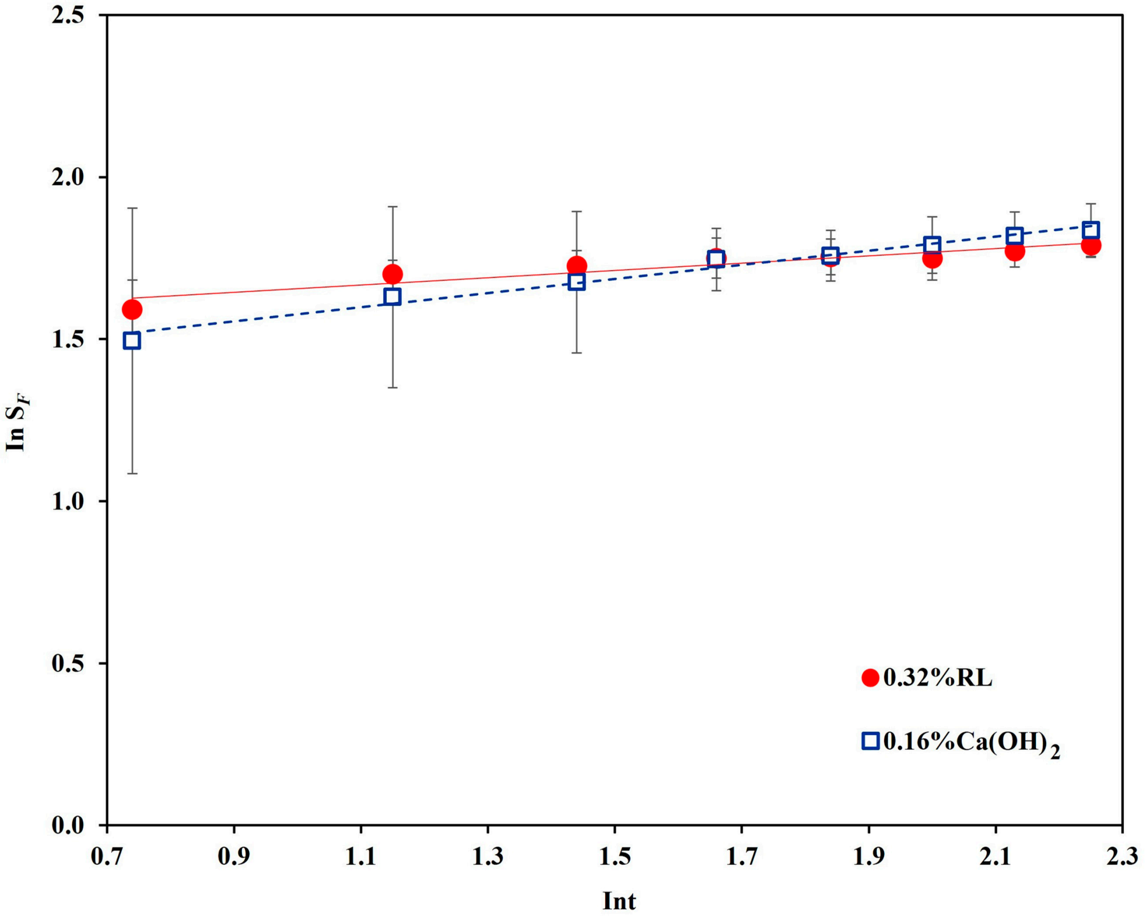

Figure S16. Relationship between ln S_F and ln t from investigation of swelling mechanisms of the cryogels.

The diffusion coefficient (D) for a Fickian diffusion could be described by equation 10 [37,44,60]:

$$S_F = 4(\frac{Dt}{\pi l^2})^{\frac{1}{2}} \quad (10)$$

where l is the radius of the cryogel, and D can be estimated from the slope of the graph plotting between S_F versus $t^{1/2}$ (Figure S17). The diffusion coefficient of water through the 0.16%$Ca(OH)_2$ cryogels (0.3±0.2 $cm^2 \cdot min^{-1}$) was higher than 0.32%RL (0.1±0.1 $cm^2 \cdot min^{-1}$) which indicates a faster diffusion rate of water in 0.16%$Ca(OH)_2$ leading to higher swelling ratio.

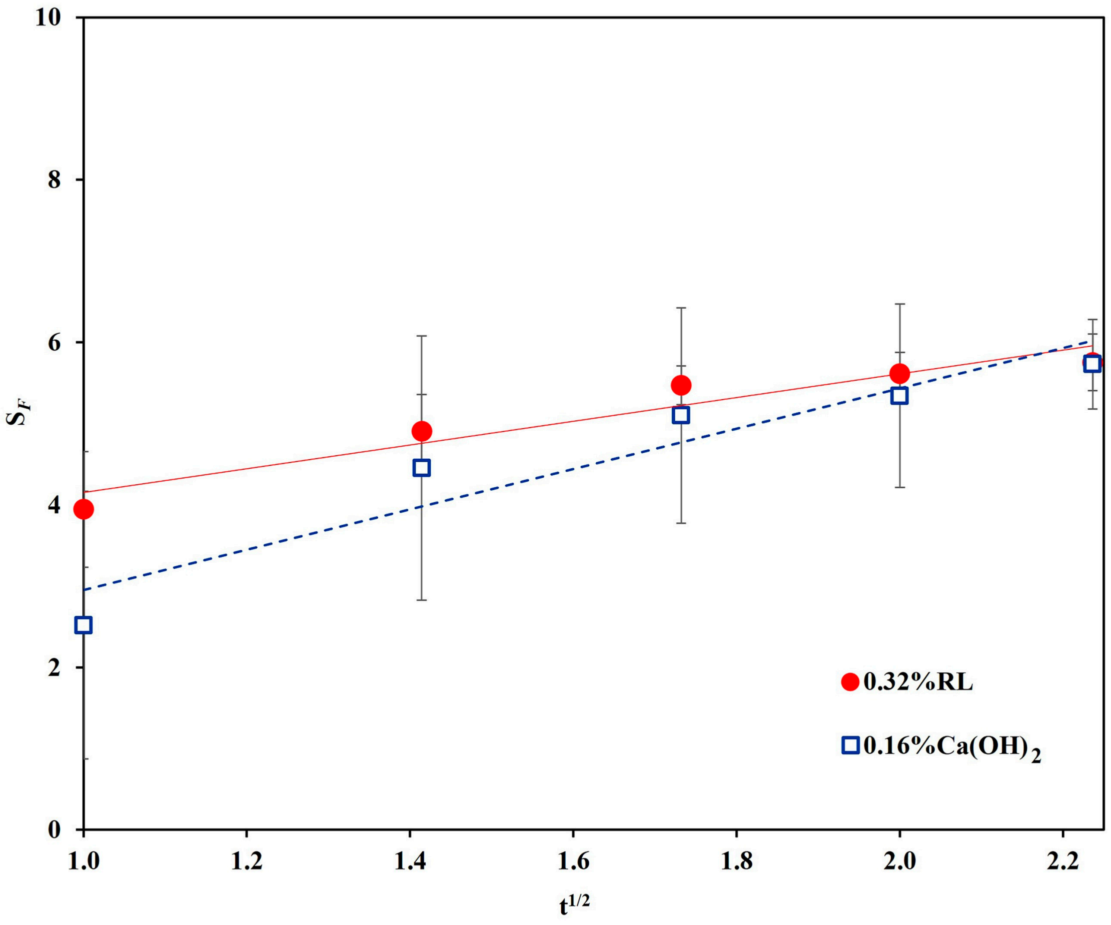

Figure S17. Diffusion curves of the prepared cryogels.

The cryogels as filters

Both cryogels were preliminarily tested as filters for 5 water samples and showed the ability to reduce the light absorption of the sample at a specific wavelength (Figure S18). The maximum adsorption at ~285-290 nm of these samples decreased in the range of 25±3 to 94±6% and 27±4 and 96±5% after filtered through the filters prepared from 0.16%$Ca(OH)_2$ and 0.32%RL, respectively.

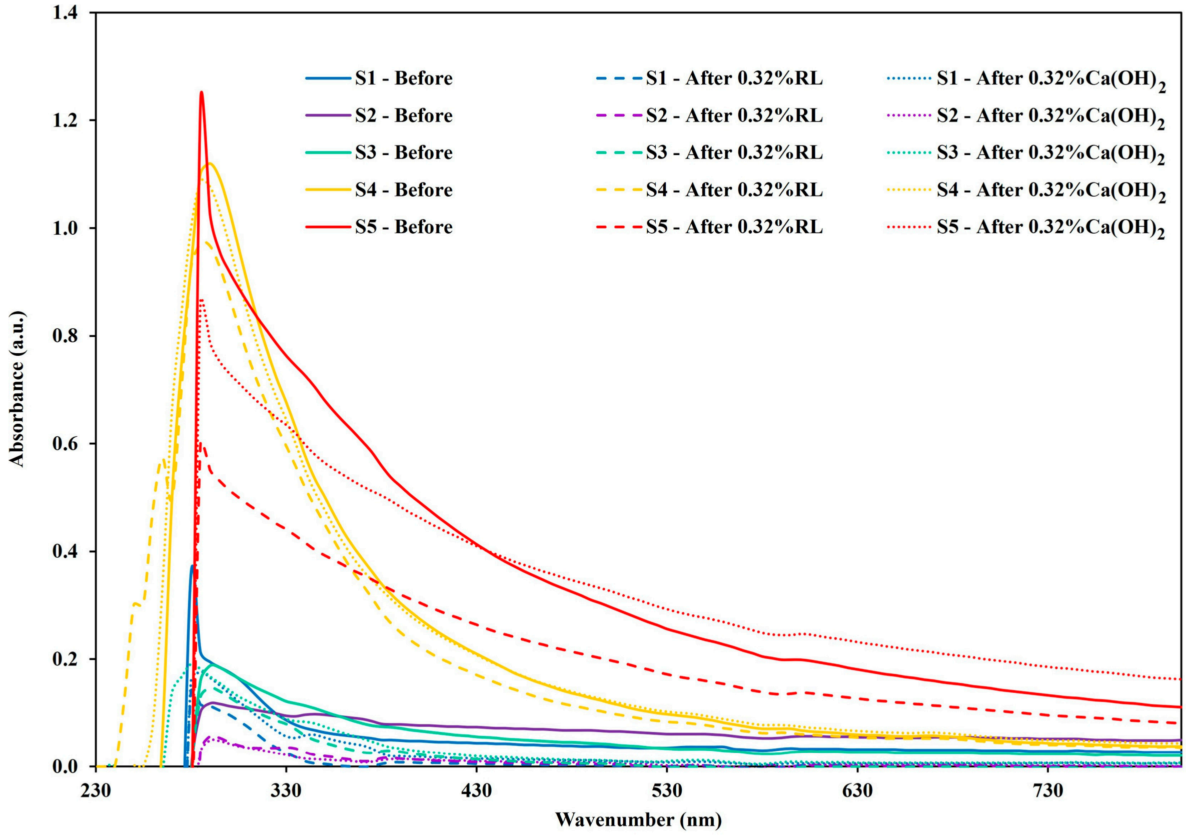

Figure S18. Absorption spectra of water samples before and after filtration using prepared filters.

Biodegradation of the cryogels

Biodegradation of the cryogels prepared from rice starch was investigated using a soil burial test as previous reported [33]. Swollen cryogels were buried at 10 cm depth under the surface of soil. They were collected at 10-day intervals and washed thoroughly to remove the soil as much as possible before weighing the remaining weight (W_b). The percentage of weight remaining was calculated using equation (11) where W_e is the weight of the swollen cryogel and W_b is the weight of the cryogel after soil burial.

$$\text{Weight remaining (\%)} = 100 \times \frac{W_b}{W_e} \quad (11)$$

The results showed the decrease of %weight remaining with the time. Both cryogels lost weight for 100% when buried in soil for 30 days due to hydrolysis and microorganisms [33].

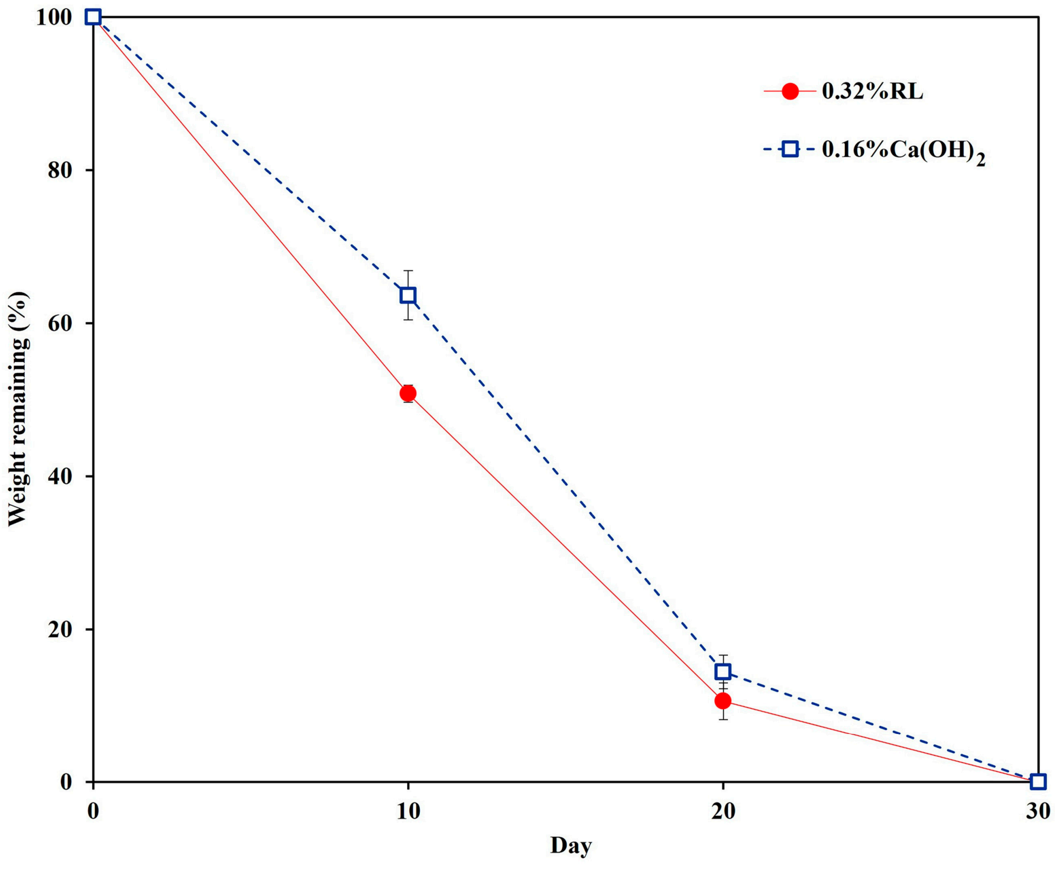

Figure S19. %Weight remaining of the prepared cryogels after burial in soil for 30 days.

References

10. Nakamatsu, J.; Torres, F.G.; Troncoso, O.P.; Min-Lin, Y.; Boccaccini, A.R. Processing and Characterization of Porous Structures from Chitosan and Starch for Tissue Engineering Scaffolds. *Biomacromolecules* **2006**, 7, 3345–3355, doi:10.1021/bm0605311.
33. Junlapong, K.; Maijan, P.; Chaibundit, C.; Chantarak, S. Effective adsorption of methylene blue by biodegradable superabsorbent cassava starch-based hydrogel. *Int. J. Biol. Macromol.* **2020**, 158, 258–264, doi:10.1016/j.ijbiomac.2020.04.247.
34. Zou, F.; Budtova, T. Tailoring the morphology and properties of starch aerogels and cryogels via starch source and process parameter. *Carbohydr. Polym.* **2021**, 255, 117344, doi: 10.1016/j.carbpol.2020.117344.
37. Jayaramudu, T.; Ko, H.U.; Kim, H.C.; Kim, J.W.; Kim, J. Swelling Behavior of Polyacrylamide-Cellulose Nanocrystal Hydrogels: Swelling Kinetics, Temperature, and pH Effects. *Materials (Basel)* **2019**, 12, 2080, doi:10.3390/ma12132080.
39. Bryant, C.; Hamaker, B. Effect of Lime on Gelatinization of Corn Flour and Starch. *Cereal Chem.* **1997**, 74, 171–175, doi:10.1094/CCHEM.1997.74.2.171.
42. Cornejo-Villegas, M.Á.; Rincón-Londoño, N.; Real-López, A.D.; Rodríguez-García, M.E. The effect of Ca2+ ions on the pasting, morphological, structural, vibrational, and mechanical properties of corn starch–water system. *J. Cereal Sci.* **2018**, 79, 174–182, doi:10.1016/j.jcs.2017.10.003.
44. Bajpai, A.K.; Shrivastava, M. Swelling kinetics of a hydrogel of poly(ethylene glycol) and poly(acrylamide-co-styrene). *J. Appl. Polym. Sci.* **2002**, 85, 1419–1428, doi:10.1002/app.10675.
46. Lozinsky, V.; Okay, O. Basic Principles of Cryotropic Gelation. *Adv. Polym. Sci.* **2014**, 263, 49–101, doi:10.1007/978-3-319-05846-7_2.
52. Jiang, X.; Jiang, T.; Gan, L.; Zhang, X.; Dai, H.; Zhang, X. The plasticizing mechanism and effect of calcium chloride on starch/poly(vinyl alcohol) films. *Carbohydr. Polym.* **2012**, 90, 1677–1684, doi:10.1016/j.carbpol.2012.07.050.
53. Boonkanon, C.; Phatthanawiwat, K.; Wongniramaikul, W.; Choodum, A. Curcumin nanoparticle doped starch thin film as a green colorimetric sensor for detection of boron. *Spectrochim. Acta A Mol. Biomol. Spectrosc.* **2020**, 224, 117351, doi:10.1016/j.saa.2019.117351.
54. Wongniramaikul, W.; Limsakul, W.; Choodum, A. A biodegradable colorimetric film for rapid low-cost field determination of formaldehyde contamination by digital image colorimetry. *Food Chem.* **2018**, 249, 154–161, doi:10.1016/j.foodchem.2018.01.021.
55. Gupta, N.V.; Shivakumar, H.G. Investigation of Swelling Behavior and Mechanical Properties of a pH-Sensitive Superporous Hydrogel Composite. *Iran. J. Pharm. Res.* **2012**, 11, 481–493, PMCID:PMC3832170.
56. Basavarajappa, S.; Perea-Lowery, L.; Aati, S.; Al-Kheraif, A.A.A.; Ramakrishnaiah, R.; Matinlinna, J.P.; Vallittu, P.K. The effect of ethanol on surface of semi-interpenetrating polymer network (IPN) polymer matrix of glass-fibre reinforced composite. *J. Mech. Behav. Biomed. Mater.* **2019**, 98, 1–10, doi:10.1016/j.jmbbm.2019.05.030.
57. Weast, R.C. *CRC Handbook of Cpresshemistry and Physics*, 66th ed.; CRC Press: Florida, USA, **1985**.
58. Chhatri, A.; Bajpai, J.; Bajpai, A.K. Designing polysaccharide-based antibacterial biomaterials for wound healing applications. *Biomatter* **2011**, 1, 189–197, doi:10.4161/biom.19005.
59. Plieva, F.M.; Andersson, J.; Galaev, I.Y.; Mattiasson, B. Characterization of polyacrylamide based monolithic columns. *J. Sep. Sci.* **2004**, 27, 828–836, doi:10.1002/jssc.200401836.
60. Murthy, P.S.K.; Yallapu, M.; Sreeramulu, J.; Raju, K.M. Semi-IPNs of starch and poly(acrylamide-co-sodium methacrylate): Preparation, swelling and diffusion characteristics evaluation. *React. Funct. Polym.* **2006**, 66, 1482–1493, doi:10.1016/j.reactfunctpolym.2006.04.010.
